# The Roles of MicroRNAs, Oncogenes, and Tumor Suppressor Gene Molecular Subtypes of Breast Cancer: Therapeutic Potential of Pharmaceutical and Natural Products

**DOI:** 10.1155/ijbc/2576658

**Published:** 2026-05-07

**Authors:** Md. Sadikuj Jaman, Md. Maniruzzaman, Md. Rokibul Hasan Bhuiyan, Md Shajedul Haque

**Affiliations:** ^1^ Department of Biochemistry and Molecular Biology, University of Rajshahi, Bangladesh, Rajshahi, ru.ac.bd; ^2^ Department of Cellular and Molecular Anatomy, Hamamatsu University School of Medicine, Hamamatsu, Shizuoka, Japan, hama-med.ac.jp; ^3^ Department of Pharmacy, Varendra University, Rajshahi, Bangladesh

**Keywords:** breast cancer, microRNA, oncogene, trastuzumab, tumor suppressor gene, ursolic acid

## Abstract

**Background:**

Breast cancer (BC) is the most common malignancy among women, with 2.3 million new cases and over 670,000 deaths annually. Despite advances in detection and therapy, relapse, metastasis, and resistance remain significant challenges. Tumor suppressor genes (TSGs) regulate abnormal cell division, whereas oncogenes, which arise from proto‐oncogenes, can inhibit TSGs and promote cancer progression. MicroRNAs further influence cellular processes in both normal and malignant states. Pharmaceutical agents such as tamoxifen and paclitaxel have demonstrated efficacy, while natural products, including baicalin and ursolic acid, show promise in modulating oncogenic pathways. Understanding BC subtypes is crucial, as they guide prognosis and enable more precise and personalized treatment strategies. These insights emphasize the complex molecular landscape and evolving approaches to BC therapy.

**Objective:**

The review was conducted using a defined set of keywords to search PubMed, Web of Science, Embase, Scopus, Google Scholar, and SciSearch databases covering publications from the inception of each database through August 2, 2025.

**Key Findings:**

This review outlines BC subtypes and their molecular features, highlighting TSGs, oncogenes, and miRNAs as therapeutic targets. Both synthetic drugs and natural compounds demonstrate potential applications in advancing BC treatment strategies.

**Conclusion:**

BC subtypes, defined by morphology and molecular features, remain vital for targeted therapy advancement. TSGs, oncogenes, and miRNAs play key roles in subtype regulation and require further study for precise detection and treatment. Synthetic drugs and natural compounds show promise as therapeutic agents. However, continued molecular research is essential to improve personalized strategies and overcome relapse, metastasis, and resistance in BC management.

## 1. Introduction

**Figure 1:**
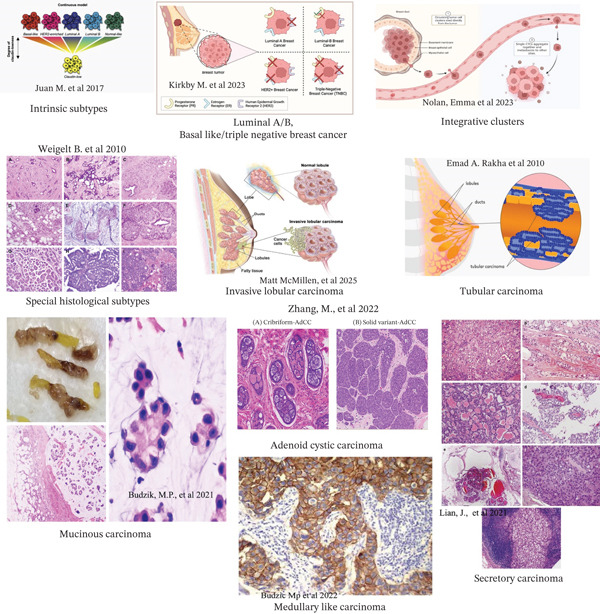
Overview of different molecularly breast cancer subtypes.

**Figure 2:**
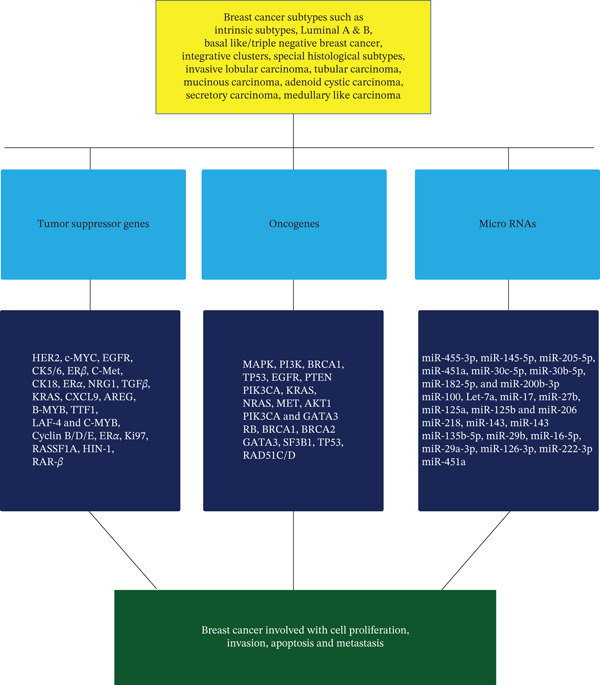
Interrelation of microRNAs with oncogenes and tumor suppressor genes in different molecularities of BC.

Breast cancer (BC) remains the most common cancer diagnosed among women worldwide, claiming the lives of approximately 67,000 individuals each year. Far from being a single disease, BC represents a diverse and heterogeneous group of conditions, reflecting its complex biological and clinical nature [1]. BC is not a uniform but a highly heterogeneous condition, even among individuals with comparable histologic stages. Patients often display distinct clinical characteristics and varied responses to combined therapies, underscoring the complexity of their management. Molecular diversity of BC compels us to explore more precise methods for identifying patients at high risk and tailoring treatment strategies accordingly. Classification of breast tumors relies on both molecular and morphological features, with morphological categorization based on site of origin and degree of invasiveness within breast tissue. This heterogeneity forms the foundation for understanding disease progression and optimizing personalized therapeutic approaches [2]. BC is classified not only by its site of origin and invasiveness but also by distinct molecular subtypes that reflect its biological diversity. Noninvasive forms such as LCIS and SCIS are confined to epithelial components of lobules or ducts without stromal invasion. IDC, the most prevalent histotype, extends beyond ducts into surrounding breast tissue and often progresses to lymphatic or systemic metastases. At the molecular level, BCs are broadly categorized into intrinsic subtypes like luminal A and B, HER2‐enriched, and basal‐like/triple‐negative based on gene expression profiles and receptor status (ER, PR, and HER2). These molecular classifications provide critical insights into prognosis and guide therapeutic strategies, underscoring the heterogeneity of BC beyond its morphological features [3]. This is depicted in Figure 1; according to molecular classification, BCs are grouped into intrinsic subtypes as luminal A and B, HER2‐enriched, basal‐like (triple‐negative), and common histologic entities (ISDC, ILC, IBC, metaplastic, apocrine, mucinous, cribriform, tubular, and neuroendocrine carcinomas) that map variably onto these molecular categories: ILC is characterized by loss of E‐cadherin and most often corresponds to luminal subtypes. IBC is a clinically aggressive presentation frequently associated with HER2‐enriched or basal‐like biology, and primary distinctions between invasive ductal and lobular carcinomas are both morphologic and genetic [4]. On the other hand, TSGs in BC fall into two functional groups: gatekeepers, which directly restrain proliferation or trigger apoptosis, and caretakers, which maintain genomic integrity and are commonly inactivated in breast tumors by mutations, deletions, promoter methylation, or enhanced proteolysis, driving tumor initiation and progression [5]. miRNAs are short noncoding RNAs that critically regulate gene expression in BC, acting as oncogenes or tumor suppressors to control proliferation, apoptosis, invasion, metastasis, and therapy response, and their stability in tissue and body fluids also makes them promising diagnostic and prognostic biomarkers [6]. OCGs in BC are mutated or overactive derivatives of proto‐oncogenes that drive uncontrolled proliferation, survival, invasion, and metastatic fitness, and common mechanisms include point mutations, gene amplification, and increased expression, with clinically actionable examples such as ERBB2/HER2 and PIK3CA that both define biology and guide targeted therapy. Their selection during tumor evolution fosters aggressive phenotypes and therapy resistance, making oncogene status central to prognosis and treatment planning [7]. Current therapy for BC commonly relies on combination cytotoxic regimens and targeted agents, yet a major clinical challenge is the emergence of resistance that reduces long‐term benefit and increases relapse risk. Resistance may be intrinsic or acquired, and it affects both conventional chemotherapy and newer targeted therapies, undermining efficacy across molecular subtypes and clinical settings [8]. Synthetic drugs are essential for subtype‐directed BC care and targeting ER/AR, HER2, PI3K/AKT/mTOR, and immune checkpoints, while combination with natural products can enhance efficacy, reduce toxicity, and help overcome resistance through complementary mechanisms such as chemosensitization, antioxidant modulation, and epigenetic effects [9]. Natural compounds such as curcumin, apigenin, and baicalin are widely consumed phytochemicals with favorable safety profiles and offer pleiotropic anticancer actions that make them promising adjuncts for treatment‐resistant BC subtypes. Although preclinical and early clinical data suggest these agents can enhance efficacy, reduce toxicity, and help overcome resistance, rigorous pharmacokinetic evaluation and randomized trials are needed to define optimal combinations, dosing, and biomarker‐guided indications [10, 11]. This is shown in Figure 2, and the aim of this study is to review past research to identify molecularly similar BCs and to examine how BC subtypes are linked to TSGs, OCGs, and miRNAs. It also evaluates the therapeutic potential of natural products that are used alone or in combination with conventional treatments.

## 2. Search Methodology

A thorough review of the literature search in PubMed, Web of Science, MEDLINE, Embase, Scopus, ScienceDirect, Google Scholar, and SciSearch databases was carried out using the following keywords: Breast cancer subtypes, Intrinsic subtypes, Luminal A & B, Basal like/Triple negative breast cancer, Integrative clusters, Special histological subtypes, Invasive lobular carcinoma, Tubular carcinoma Mucinous carcinoma, Adenoid cystic carcinoma, Secretory carcinoma, Medullary like carcinoma, micro RNA, oncoprotein, tumor suppressor gene, and Pharmaceutical agents like Hormonal, chemotherapeutics, targeted drug, and natural product. About 1200 papers for the BC molecular subtype, 856 researches for BC treatment with pharmaceutical drugs, and 320 findings associated with BC treatment with natural products have been listed. Many duplicate studies, only abstracts showing, and non‐English studies were eliminated from a total of 756 studies. The remaining titles and abstracts were checked, and studies not relevant to the topics were excluded, along with papers for which no full text could be accessed. The titles and abstracts of the remaining papers were examined, and studies that were unrelated to the subject and publications for which there was no full text available were both eliminated. In total, 250 studies matched our inclusion criteria, and their findings are described in this review. The majority of them focused on the molecular subtype of BC study, BC subtype related to miRNA, oncoprotein, tumor suppressor gene, and several pharmaceutical and natural drug applications on BC subtype.

### 2.1. Necessity of Discussion of MicroRNAs, Oncogenes, and Tumor Suppressor Gene′s Role in BC Subtypes

Oncogenes, which are modifications of proto‐oncogenes involved in normal regulation of cell development and proliferation, are the first cancer genes to be thoroughly examined molecularly. When these genes are altered, cell growth and proliferation occur, which are known as gain‐of‐function. These genes are in charge of initiating cell division when the cell is at rest. It could be explained that they are like pressing the accelerator on a car. TSGs prevent cell development and proliferation, whereas oncogenes encourage it [12]. However, miRNAs use a post‐transcriptional process that includes translation suppression and miRNA degradation to control gene expression. Specifically, miRNAs enable the removal of poly (A) tail from signals to which they are partly complementary, hence speeding up mRNA turnover. 5/decapping is triggered by deadenylation and consequent loss of poly (A) binding protein, which encourages exonucleolytic digestion from the 5/end. The cap binding affinity of the miRNA binding protein provides an explanation for the entire translation repression mechanism. When the cap is hidden, it is unable to attach to the eIF4E initiation factor. A small number of proteins mediate mRNA degradation and translational repression [13]. The RNA‐induced silencing complex, which transports those little RNAs to complementary locations inside mRNA, includes several of these proteins as members. miRNA typically uses improper base pairing to interact with a target. miRNA can have several targets, and the selectivity of miRNA targeting may be influenced by biological variables linked to the RISC complex. The influence of these miRNA targets on gene expression can be greatly increased despite the fact that many of them are involved in different signaling pathways. Thus, miRNA is crucial for the regulation of genes. We are aware that there are various subtypes of BC. Numerous studies on the various forms of BC and their relation with miRNA, oncogenes, or tumor suppressor genes have already been published [14]. We are aware that there are six main subtypes of BC. Tumors enriched with HER2, luminal A/B, basal‐like, normal‐like, and claudin‐low subtypes are the six main subtypes. While luminal B presents ER, PR, and HER2, it may be distinguished from luminal A by its strong Ki67 staining, which indicates a faster rate of proliferation. Luminal A is recognized by ER, PR, BCL‐2, and the absence of HER2. ADP ribose polymerase‐1 inhibitors are developed by basal‐like BCs, which have a poor prognosis and do not respond to ER, PR, or HER2. The normal‐like subtype lacks CK2 and EGFR expression but has high epithelial gene expression. E‐cadherin is a calcium‐dependent glycoprotein that promotes cell‐to‐cell adhesion, while claudin is a tight junction protein [15]. On the other hand, this subtype is likewise linked to miRNA, and numerous miRNAs have already been investigated. For example, ER, PR, HER2, and TNBCs overexpressed miR‐21, miR‐210, and miR‐221. However, these subtypes exhibit underexpression of miR‐10b, miR‐145, miR‐205, and miR‐122a. Eight miRNAs, including let7c, miR‐125b, miR‐126, miR‐127‐3P, miR‐143, miR‐145, miR‐146‐5P, and miR‐199a‐3P, are elevated in basal‐like subtypes, while the majority of miR‐200c and miR‐429 are luminal. Approximately 664 miRNAs were examined, and 116 are expressed in normal breast epithelium. Furthermore, myoepithelioma has higher levels of miR‐126, miR‐127, miR‐143, miR‐145, and miR‐199 than luminal and basal‐like subtypes. Malignant myoepithelioma had lower levels of miR‐200c and miR‐429 than luminal and basal cancers, such as BC. Researchers found that miR‐520g, miR‐377, miR‐527‐518a, and miR‐520f‐520c are HER2‐specific, while miR‐342, miR‐299, miR‐217, miR‐190, miR‐135b, and miR‐218 are ER‐specific. Subsequent investigation showed that while miR‐520g expression was downregulated in ER‐ and PR‐positive tumors, miR‐342 expression was highest in ER‐ and human epidermal growth factor receptor 2‐positive (HER2+) luminal B tumors and lower in triple‐negative tumors [16]. The most crucial subjects include miRNA roles of oncogenes and tumor suppressor genes and crucial elements in the identification of BC subtypes. The purpose of this review is to gather specific data regarding various subtypes of BC and determine how oncogenes, tumor suppressor genes, and miRNAs interact.

### 2.2. Intrinsic Subtype of BC

BC′s intrinsic subtype is made up of several subtypes with unique morphologies. Although there is a lot of overlap between these subtypes that were discovered utilizing various gene panels, the introduction of new subtypes has complicated our knowledge of breast tumor heterogeneity. This is shown in Table 1 and Figure 1 [33]. Moreover, technological advancement made it possible for researchers to compare a vast number of samples in order to gain a deeper understanding of the nature of the disease. Gene expression–based ranking has joined conventional criteria used to classify BC. MammaPrint, the genomic grade index, contributed a better understanding of signaling pathways controlling the development, maintenance, and growth of tumors by offering insightful information on tumor biology. HER2, ER, IGF1R, PI3K/AKT, mTOR, AMPK, and angiogenesis pathways are among the mechanisms that researchers currently understand better [34]. High enrichment for epithelial‐to‐mesenchymal transition markers, immune response genes, and characteristics of cancer stem cells, as well as poor or missing expression of luminal differentiation markers, are characteristics of claudin‐deficient malignancies. In addition to this, their response rate to conventional preoperative treatment falls somewhere between that of luminal and basal‐like cancers. It is interesting to note that a number of genetically modified mouse models and a collection of widely used BC lines exhibit the claudin‐low phenotype [35]. Additionally, creating a risk model that includes gene expression–based intrinsic subtypes of basal‐like, HER2‐enriched, and luminal A and B in order to improve upon present criteria for BC prognosis and chemotherapy benefit prediction. Then, 97% negative predictive value for the PCR intrinsic subtype model forecasted the effectiveness of neoadjuvant chemotherapy [17]. Also, after 3 years, luminal A showed a gradual rise in risk that peaked and then leveled off. The majority of luminal B relapses occurred within the first 5 years [18]. Furthermore, BC can be categorized into various groups according to their biological characteristics, histological characteristics, and/or clinical behavior. It will make it easier to identify treatment resistance mechanisms, investigate tumor evolution, and find new drivers. Prototypical patterns of single‐nucleotide variations are revealed by this categorization of breast tumors into integrative clusters, which are linked to different clinical trajectories and treatment responses [36]. Besides, BC intrinsic subtypes are separate biological entities that exhibit particular patterns of natural gene expression. In order to prevent more misunderstanding and patient harm, researchers give readers a historical summary of intrinsic discovery and clinical application, highlighting biological and technical distinctions for detection and suggesting clear and straightforward nomenclature for subtyping [37]. What is more, identifying ISs of BC that are represented in a group based on the quantitative expression of HER2 and HR. There is still a great deal of disagreement between IS and clinical assay–defined subsets. The ideal HR IHC cut point for detecting basal‐like BC was less than 1% in accordance with recommendations of the College of American Pathologists and the American Society of Clinical Oncology. Due to their genetic diversity, tumors with borderline HR staining would need further tests to elucidate the underlying biology [38], whereas molecular alterations that take place while BC spreads are still not fully understood. Luminal/HER2‐negative cancers developed a luminal B or HER2 profile after metastatic spread, which most likely reflected tumor evolution or acquisition of estrogen independence [39]. On the other hand, it is unclear whether biological heterogeneity in HER2+ BC affects clinical outcomes. The HER2‐enriched subtype was more common in cHER2‐positive BC than clinical HER2‐negative BC, while luminal A and basal‐like subtypes were less common. In the absence of HER2 targeting, cHER2 positivity has no effect on patient survival and does not result in significant alterations in expression of downstream signaling pathways when ISs are considered [40]. Besides, BC is a diverse illness with a range of genetic, histological, and clinical manifestations. It is crucial to determine whether gene expression profiling could enhance the current conventional immunohistochemistry‐based clinical subtyping approach and whether this approach has the potential to be implemented in clinical practice, even though mounting evidence suggests that ISs offer clinically relevant information beyond clinical surrogates [41].

**Table 1:** Studies of genetic assays for different BC subtypes.

Types	Appearance	Reference
Intrinsic subtypes	97%	[17]
Intrinsic subtypes	14%	[18]
Luminal	90%	[19]
Luminal A and B	70%	[20]
Basal‐like/triple‐negative breast cancer	15%–25%	[21]
Integrative clusters	67%	[22]
Special histological subtypes	9.8%–16.8%	[23]
Special histological subtypes	25%	[24]
Invasive lobular carcinoma	90%	[25]
Invasive lobular carcinoma	68.8%	[26]
Tubular carcinoma	50%–70%	[27]
Tubular carcinoma	71.2%	[28]
Mucinous carcinoma	64%	[29]
Adenoid cystic carcinoma	0.1%	[30]
Secretory carcinoma	15.4%	[31]
Medullary like carcinoma	36%	[32]

### 2.3. Key Driver Mutations in Intrinsic Subtype

The IS of BC is influenced by oncogenes. Certain patterns of metastatic spread were linked to distinct intrinsic molecular subtypes. Luminal tumors were more likely to develop bone‐only metastases, basal‐like tumors were linked to a higher risk of lung metastases, and HER2‐enriched tumors were more likely to develop brain metastases than other subtypes. This is shown in Table 2 [42]. Moreover, the relationship between IS‐specific serum HER2 levels at diagnosis and clinicopathologic characteristics and DFS in individuals with operable BC. Fifty‐two patients (11.9%) had high sHER2 levels, while 111 patients (25.5%) had HER2 overexpression in their tumor tissue. BC patients, particularly those with HR+/HER2−, HR+/HER2+, and HR−/HER+ subtypes, and sHER2 level at diagnosis are valuable prognostic indicators [43]. Hence, in a large, well‐annotated patient cohort, the relationship between intrinsic subtype and clinical outcome was evaluated by analyzing genomic data from HER2+ cancers. The Prosigna algorithm was used to classify most clinically characterized HER2+ tumors as either luminal or HER2‐enriched. Since adjuvant trastuzumab is beneficial for many cancers classified as luminal A or B, IS alone cannot replace traditional histological examination of HER2 status [44]. However, important subjects for IS of BC are tumor suppressor genes. Only a small percentage of the numerous studies we have looked at can be explained. Breast tumor classification and therapy guidance have been based on hormone receptor status. Heterogeneity is also visible in these molecular pictures, which are used to classify breast tumors using gene expression profiling. Selective clonality within growing neoplasms is a complicated element in the categorization of BC. A subclone of cancer cells that triggers a recurrence of disease may not always be reflected in the molecular profiles of a heterogeneous initial tumor. This is shown in Table 3 [128]. Furthermore, IHC measurement of p53 accumulation in intrinsic subtypes, St. Gallen consensus subgroups, and LNN‐BC was used to evaluate its prognostic significance. DFS was significantly impacted by tumor size, p53, Ki67, ER, and PR. According to this characteristic, PR‐negative and p53‐positive were independent predictive factors in multivariate analysis. LNN‐BC power provides extraprognostic data for the St. Gallen consensus and ISs [87]. Besides, TP53 mutation and DNA methylation are used as intrinsic BC subgroups for better survival prediction and characterization. Then, 38% of sequenced samples had a TP53 mutation, which primarily impacted TNBC patients (87%). According to the COX analysis, RUNX3 methylation and TN status are independent predictors of OS in BC. Predicting BC outcomes, where a combination of two to three epigenetic indicators may be a useful approach [88]. Consequently, BC is often associated with activation of PI3K and MAPK pathways. Since single proteins are known to provide insufficient information, it is crucial to analyze numerous proteins as readouts for pathway activation in ER/HER2− BC. Additional IS of primary BC were identified that were obtained from postmenopausal patients [89]. Therefore, ER+ BC cell lines using shRNA expression vectors brought PTEN and LC3I/II. In resistance, human BC samples PTEN and LC3I and II were shown to be downregulated. Transtuzumab resistance was elevated in BC cells when PTEN and PTEN+ LC3I/II were knocked down with shRNA. Through autophagy abnormalities, PTEN facilitates the establishment of primary trastuzumab resistance in BC [90]. Even though early BCs that are ER+/HER‐positive and do not have a BRCA1/2 mutation may have a high HRD score. Subgroup of HR+/HER2− BC that is BRCA proficient and has a high HRD score. Lastly, BRCA‐proficient HRD‐low tumors showed markedly immune, mutational, and tumor molecular signature landscapes [91].

**Table 2:** Studies of various oncogene activities on different BC subtypes.

Molecular subtype of breast cancer	Oncogene names	Functions	Reference
Intrinsic subtype	HER2	Downstream signaling pathway	[42]
Intrinsic subtype	HER2	Useful prognostic factor	[43]
Intrinsic subtype	HER2	Trastuzumab application	[44]
Luminal A	c‐MYC	Therapeutic use	[45]
Luminal subtype	c‐MYC	Therapeutic target	[46]
Luminal subtype	Cyclin D1		[47]
Basal‐like/triple‐negative breast cancer	EGFR, CK5/6, ER*β*, and C‐Met	Prognostic purpose	[48]
Basal‐like/triple‐negative breast cancer	CK18 and ER*α*	Therapeutic target	[49]
Basal‐like/triple‐negative breast cancer	NRG1 and HER2	Inhibitor	[50]
Basal‐like/triple‐negative breast cancer	c‐MYC and TGF‐*β*	Metastasis blocker	[51]
Basal‐like/triple‐negative breast cancer	Cyclin E/CDK	Therapeutic target	[52]
Integrative clusters	c‐MYC and KRAS	Biomarker	[53]
Integrative clusters	CXCL9, AREG, B‐MYB, TTF1, LAF‐4, and c‐MYB	Biomarker	[54]
Integrative clusters	HER2	Therapeutic target	[55]
Integrative clusters	Cyclin B/D/E and c‐MYC	Therapeutic target	[56]
Integrative clusters	Cyclin E	Therapeutic target	[57]
Integrative clusters	ER*α*	Biomarker	[58]
Special histological subtypes	HER2	Therapeutic and prognostic marker	[59]
Special histological subtypes	HER2	Prognostic factor	[60]
Special histological subtypes	CCND1	Prognostic factor	[61]
Special histological subtypes	c‐MYC	Biomarker	[62]
Invasive lobular carcinoma	c‐MYC	Therapeutic target	[63]
Invasive lobular carcinoma	c‐MYC	Tumor progression	[64]
Invasive lobular carcinoma	Cyclin D1 and Ki97	Suppressor	[65]
Invasive lobular carcinoma	RASSF1A, HIN‐1, RAR‐*β*, and cyclin D2	Indicator of etiological and clinical behavior	[66]
Invasive lobular carcinoma	ER*α*	Prognosis factor	[67]
Tubular carcinoma	HER2	Prognostic and therapeutic application	[68]
Tubular carcinoma	HER2	Biomarker	[69]
Tubular carcinoma	Cyclin D	Cell cycle regulator	[70]
Tubular carcinoma	Cyclin E	Proliferation	[71]
Mucinous carcinoma	HER2	Therapeutic target	[72]
Mucinous carcinoma	HER2	Biomarker	[73]
Mucinous carcinoma	c‐MYC	Cell cycle regulator	[74]
Mucinous carcinoma	HRAS	Cell cycle regulator	[75]
Adenoid cystic carcinoma	c‐MYC	Prognostic and therapeutic target	[76]
Adenoid cystic carcinoma	c‐MYC	Cell cycle regulator	[77]
Adenoid cystic carcinoma	HRAS	Biomarker	[78]
Adenoid cystic carcinoma	ER*α*	Therapeutic target	[79]
Secretory carcinoma	HER2	Prognosis and therapeutic target	[80]
Secretory carcinoma	c‐MYC	Wnt/*β*‐catenin pathway	[81]
Secretory carcinoma	c‐MYC, c‐erbB‐2, and Ki67	Prognostic factor	[82]
Medullary like carcinoma	c‐MYC	Prognostic and therapeutic target	[83]
Medullary like carcinoma	HER2	Clinical behavior study	[84]
Medullary like carcinoma	c‐MYC and cyclin D	Prognostic factor	[85]
Medullary like carcinoma	Cyclin E	Prognostic marker	[86]

**Table 3:** Studies of various tumor suppressor gene activities on different BC subtypes.

Molecular subtype of breast cancer	Tumor suppressor gene names	Functions	Reference
Intrinsic subtype	TP53	Prognostic power	[87]
Intrinsic subtype	TP53	Prognostic factor	[88]
Intrinsic subtype	PTEN, AKT, MAPK, and PI3K	Signaling pathway	[89]
Intrinsic subtype	PTEN	Autophagy signal transduction	[90]
Intrinsic subtype	BRCA1/BRCA2	Genomic, transcriptomic, and immunic landscape	[91]
Luminal subtype	BRCA1/BRCA2	Caretaker	[92]
Luminal subtype	BRCA1/BRCA2	Caretaker	[93]
Basal‐like/triple‐negative breast cancer	BRCA1, TP53, EGFR, and PTEN	Therapeutic target	[94]
Basal‐like/triple‐negative breast cancer	PTEN and TP53	Therapeutic target	[95]
Basal‐like/triple‐negative breast cancer	BRCA1, TP53, and PIK3CA	Prognostic factor	[96]
Basal‐like/triple‐negative breast cancer	KRAS, NRAS, MET, and AKT1	Biomarker for targeted therapy	[97]
Basal‐like/triple‐negative breast cancer	BRCA1	Biomarker	[98]
Integrative cluster breast cancer	TP53, PIK3CA, and GATA3	Prognostic factor	[99]
Integrative cluster breast cancer	TP53	Tumor suppressor	[100]
Integrative cluster breast cancer	PTEN	Immunotherapy target	[101]
Integrative cluster breast cancer	BRCA1/2		[102]
Special histological subtypes	RB and BRCA1	Therapeutic target	[103]
Special histological subtypes	BRCA2 and RB	Biomarker	[104]
Special histological subtypes	P53	Prognostic factor	[105]
Special histological subtypes	P53	Therapeutic target	[106]
Invasive lobular carcinoma	TP53 and BRCA1/2	Biomarker	[107]
Invasive lobular carcinoma	BRCA1, BRCA2, TP53, and CDH1	Biomarker and breast cancer screening	[108]
Invasive lobular carcinoma	RB	Prognostic factor	[109]
Invasive lobular carcinoma	PTEN	Therapeutic target	[110]
Tubular carcinoma	RB	Clinical application	[111]
Tubular carcinoma	PTEN	Suppression	[112]
Tubular carcinoma	BRCA1 and BRCA2	Biomarker	[113]
Mucinous carcinoma	GATA3, SF3B1, and TP53	Cell signal inhibitor	[114]
Mucinous carcinoma	TP53	Survival marker	[115]
Mucinous carcinoma	BRCA2	Biomarker	[116]
Mucinous carcinoma	PTEN	Biomarker	[117]
Adenoid cystic carcinoma	PTEN	PI3K/AKT signal blocker	[118]
Adenoid cystic carcinoma	PTEN and TP53	Biomarker and therapeutic target	[119]
Adenoid cystic carcinoma	BRCA1/2	Pathogenic marker	[120]
Secretory carcinoma	BRCA1/2, TP53, and RAD51C/D	Understanding secretory carcinoma	[121]
Secretory carcinoma	BRCA1/2	Cell cycle study	[122]
Secretory carcinoma	PTEN and TP53	Understanding signaling pathway and tumoral development	[123]
Medullary like carcinoma	TP53	Prognostic factor	[124]
Medullary like carcinoma	TP53	Biomarker	[125]
Medullary like carcinoma	PTEN	Therapeutic target	[126]
Medullary like carcinoma	BRCA1	Cell cycle regulator	[127]

### 2.4. miRNA Signatures and Metastasis in Intrinsic Subtype

miRNA is a fascinating and significant topic in IS of BC. Although many miRNAs have been identified for BC, very few are addressed in BC molecular subtypes. Such as compared to HR‐positive BC, TNBC had significantly higher levels of miR‐455‐3p and miR‐196a‐5p, while miRNA microarray research showed that miR‐425‐5p expression was higher in TNBC cell lines MDA‐MB‐231 and MDA‐MB‐468. TNBC cells′ capacity to invade and migrate was enhanced by siRNA that targeted the E124 gene. This is shown in Table 4 [129], whereas IBC had significantly lower levels of miR‐145‐5p, miR‐205‐5p, and miR‐451a than DCIS did overall, as well as ER± subgroups. In the same tumors, their expression levels were considerably lower in the invasive component than in the in situ components. In DCIS with a HER2+ status and high Ki67 proliferation index, miR‐145, miR‐205, and miR‐451 frequently show decreased expression levels [130].

**Table 4:** Studies of various microRNA activities on different BC subtypes.

Studies of breast cancer cell line	Name of microRNAs	Functions	Reference
Intrinsic subtype	miR‐455‐3p	Promote invasion and migration	[129]
Intrinsic subtype	miR‐145‐5p, miR‐205‐5p, and miR‐451a	Microinvasion	[130]
Luminal subtype	miR‐30c‐5p, miR‐30b‐5p, miR‐182‐5p, and miR‐200b‐3p	Prognostic value	[131]
Luminal subtype	miR‐100	Therapeutic use	[132]
Luminal subtype	microRNAs	Prognostic, predictive, and diagnosis factor	[133]
Basal‐like/triple‐negative breast cancer	miR‐135b	Prognostic factor	[134]
Basal‐like/triple‐negative breast cancer	miR‐126‐3p	Prognostic factor	[135]
Basal‐like/triple‐negative breast cancer	miR‐143/145	Therapeutic marker	[136]
Basal‐like/triple‐negative breast cancer	miR‐134, miR‐146a, and miR‐181b	Therapeutic marker	[137]
Basal‐like/triple‐negative breast cancer	miR‐126‐3p	Tumor suppressor and therapeutic marker	[138]
Integrative cluster breast cancer	miR‐148b and miR‐133a	Biomarker	[139]
Integrative cluster breast cancer	miR‐127	Therapeutic target and marker	[140]
Integrative cluster breast cancer	miR‐342	Caretaker	[141]
Integrative cluster breast cancer	miR‐135b‐5p	Therapeutic target	[142]
Special histological subtypes	Let‐7a, miR‐17, miR‐27b, miR‐125a, miR‐125b, and miR‐206		[143]
Special histological subtypes	miR‐218	Involvement with prognosis, endocytosis, and gap junction pathways	[144]
Special histological subtypes	miR‐143	Tumor immune microenvironment, anticancer, and suppression	[145]
Special histological subtypes	miR‐143	Suppressor, regulator, and predictor	[146]
Special histological subtypes	miR‐34a	Inhibits cancer cell survival and therapy resistance by directly targeting HDAC1 and HDAC7	[147]
Invasive lobular carcinoma	miR‐29b	Promotes apoptosis through BCL‐2 inhibition; enhances p53‐mediated tumor suppression	[148]
Invasive lobular carcinoma	miR‐16‐5p, miR‐29a‐3p, miR‐126‐3p, and miR‐222‐3p	Housekeeper	[149]
Invasive lobular carcinoma	miR‐451a and miR‐5571‐3p	Therapeutic marker	[150]
Invasive lobular carcinoma	miR‐145, ‐182, ‐21, ‐27a, ‐29b, and ‐34a	Biomarker	[151]
Invasive lobular carcinoma	miR‐146a‐5p, miR‐126‐5p, miR‐122‐5p, miR‐16‐5p, miR‐142‐3p, miR‐223‐3p, miR‐103a‐3p, miR‐221‐3p, miR‐21‐5p, and miR‐30d‐5p	Prognostic and predicting marker	[152]
Invasive lobular carcinoma	miR‐21, miR‐155, miR‐27a, miR‐205, miR‐145, and miR‐320a	Therapeutic marker	[153]
Tubular carcinoma	miRNA‐127 and miRNA‐138	Therapeutic marker	[154]
Tubular carcinoma	miR‐145	Suppression and angiogenesis	[155]
Tubular carcinoma	miR‐218	Angiogenesis	[156]
Tubular carcinoma	miR‐342‐36p	Therapeutic target	[157]
Mucinous carcinoma	miR‐217	Therapeutic target	[158]
Mucinous carcinoma	miR‐143 and miR‐224‐	Caretaker	[159]
Mucinous carcinoma	miR‐145	Molecular target	[160]
Adenoid cystic carcinoma	miR‐520g	Therapeutic target	[161]
Adenoid cystic carcinoma	miR342 and miR‐520	Therapeutic target	[162]
Adenoid cystic carcinoma	miR‐125a and miR27b	Therapeutic target	[163]
Adenoid cystic carcinoma	miR‐143	Cell cycle blocker	[164]
Adenoid cystic carcinoma	miR‐145 and miR‐199‐3p	Biomarker	[165]
Secretory carcinoma	miR‐127‐3p	Therapeutic target	[166]
Secretory carcinoma	miR‐520g	Prognostic factor	[167]
Secretory carcinoma	miR‐520/373 family. miR‐520c and miR‐373	Contribute to progression, invasion, and inflammation	[168]
Secretory carcinoma	miR‐126	Cancer study	[169]
Medullary like carcinoma	miR‐143 and ‐145	Biomarker	[170]
Medullary like carcinoma	miR‐155, miR‐15/107/182, miR‐146a, miR‐146‐5p, miR‐342, and miR‐21	Cell cycle study	[171]
Medullary like carcinoma	miR‐199a‐3p	Prognostic and therapeutic study	[172]
Medullary like carcinoma	miR‐622	Biomarker	[173]
Medullary like carcinoma	miR‐145‐5p, miR‐191‐5p, miR‐21‐5p, miR‐222‐3p, and miR‐39	Biomarker	[174]

### 2.5. Luminal BC

Luminal BC is a unique subtype because it has ER/PR receptors that are HER2 negative. Then, 70% of cases are possible, and its presence is frequent. Luminal A and B subtypes are further classifications for luminal malignancies. Determining prognoses is associated with the diagnosis of tubular, cribriform, and lobular carcinomas. This is shown in Table 1 and Figure 1 [19]. However, gene expression profiling can be used to classify IBC into prognostic and predictive molecular subtypes, including luminal BC. Based on levels of mRNA gene expression, IBC can be classified into molecular subtypes. The prognosis of patients with luminal BC and their responses to treatment can be predicted using this molecular subtyping, with risk ratings based on gene expression profiling [175]. Moreover, luminal BCs are classified into HER2− luminal A and HER2+ luminal B subtypes. Biomarkers are essential for clinical management of invasive BC because they are crucial for functional characterization, prognostication, and therapeutic prediction. Emerging technologies, such as liquid biopsies that collect and analyze CTCs and ctDNA, offer a personalized approach to predicting oncology treatment [20]. Furthermore, the St. Gallen International Expert Consensus 2011 has recommended a new classification system for BC. Imaging cytometry and FISH were used to measure DNA ploidy and CIN. There were 336 tumors in this series, including 207 aneuploids and 129 diploids [176].

### 2.6. Key Driver Mutations in Luminal BC

Numerous studies have addressed oncogenes for luminal BC. Tumors were divided into three groups based on the complicated arm aberration index and DNA copy number data. According to PAM50 subtyping, mRNA expression profiles separated malignancies into five molecular groupings, whereas miRNA expression–based clustering identified four subgroups. Identification of six integrated subtypes demonstrates that there are clearer subtype subdivisions and validates the heterogeneity of BC. Increase awareness of luminal differences, important information may be added by a subclass. This is shown in Table 2 [45]. However, about 15% of BCs have MYC amplified, and this is linked to a poor prognosis. Such as grade and basal‐like BCs were substantially linked to high MYC and c‐MYC expression. c‐MYC was linked to ATM, cyclin B1, PIK3CA, and Ki67 in luminal A tumors. Otherwise, only p16 expression and cyclin E were positively correlated with c‐MYC in basal‐like tumors [46]. Additionally, a subtype of luminal BC with poor survival was associated with deletions of PPP2RA. This subgroup showed amplification of a chromosomal region that contained gene CCND1, which codes for cyclin D1. The PPP2R2A/cyclin D1 phenotype is substantially linked to lower OS and DFS. Subgroups of BC patients with luminal‐like characteristics are defined by a combination of elevated cyclin D1 expression by IHC and altered PPP2R2A [47]. However, in luminal BC, TSGs are essential for the initiation and spread of the disease. These genes, normal processes for DNA repair and cell proliferation, can be interfered with by mutations and inactivation. Since BC and BRCA are essential, but BRCA mutation transmission may occur in BC patients, and around 21.8% of women are detected. This is shown in Table 3 [92]. Whereas in BC patients, oncofertility plays a significant role in the first phase, and 58 women were seen, whereas in the second phase, about 41 women were seen. Within 24 years, there was no discernible difference in mean age at diagnosis. Most of them had Stage II luminal‐type diagnoses and were married. Reproductive physicians′ comprehension of the oncofertility concept was the primary reason for observation of oncofertility practices [93].

### 2.7. miRNA Signatures and Metastasis in Luminal BC

Endocrine therapy is one of the most popular and successful adjuvant treatments for BC subtypes. miRNA may be predictive and/or prognostic biomarkers in this context, as they have been linked to many pathways underlying endocrine resistance and sensitivity. Clinical benefit from endocrine therapy was found to be independently predictive by miR‐30c‐5p, miR‐30b‐5p, miR‐182‐5p, and miR‐200b‐3p. Following endocrine therapy, luminal BC patients showed independent value for disease recurrence for miR‐182‐5p and miR‐200b‐3p. This is shown in Table 4 and Figure 2 [131]. Moreover, luminous chemotherapies do not help BC, but hormonal treatments typically do. The role of miR‐100 in BCs′ susceptibility to paclitaxel therapy. Luminal A subtypes showed more downregulation than others. Overall survival was decreased for patients with a lower expression level of miR‐100 [132]. However, the predictive and prognostic significance of miRNAs in luminal A BC is that a changed miRNA expression profile can identify specific molecular subtypes of BC, such as HER2, luminal A/B, and TNBC. Due to differentiation between cancer and healthy samples, a potential strategy in the realm of personalized medicine is early detection and categorization of BC subtypes using miRNA expression profiles [133].

### 2.8. Basal‐Like/Triple‐Negative BC

There is significant overlap between distinct molecular classifications of BC, TNBC, and the basal‐like subtype. Subjects′ tumor sections were immunohistochemically labeled for basal markers EGFR, c‐Kit, and 34*β*E12. Then, 22% of all BCs were TNBC. Due to the strong correlation between stage and family history of cancer, BL and NBL subtype proportions were the same. This is shown in Table 1 and Figure 1 [21]. However, to explain hormone‐responsive BCs, basal‐like, triple‐negative, and BRCA1‐positive tumors appear to share common clinical, pathological, and genetic characteristics. Molecular pathologies of these aggressive tumors have been better understood thanks to gene profiling studies of this diverse subset, which have also helped identify potential therapeutic targets [177]. Hence, TNBC is a diverse illness, and to create efficient treatment plans, tumor subtypes must be recognized and categorized in personalized medicine. Four main classes of TNBC are luminal androgen receptor, immune‐enriched, mesenchymal, and basal‐like [178].

### 2.9. Key Driver Mutations in Basal‐Like/Triple‐Negative BC

TNBC and BLBC are two types of BC that have similar poor prognoses, and Er*β*′s role in disease pathophysiology and treatment has remained debatable. Using CK5/6 and EGFR as markers, basal‐like and non‐basal‐like cancer and c‐Met or Er*β* expression levels and prognosis in TNBC patients were then ascertained. EGFR was 40.2%, and CK5/6 was 31.5%. TNBC cases were categorized as basal‐like BC because 55.1% tested positive for either EGFR or CK5/6. This is shown in Table 2 [48]. However, SHP2 inhibition in BTBC cells suppresses mesenchymal and invasive properties while inducing luminal‐like epithelial morphology. This process is known as the basal‐to‐luminal transition. Expression of the luminal marker CK18 and lack of the basal marker alpha smooth muscle actin were used to demonstrate the presence of BLT [49]. Furthermore, MCF10A generated with HER2 overexpression was used to assess and validate pertuzumab′s capacity to inhibit NRG1*β*‐induced 3D development. ERBB3, often referred to as HER3, is a tyrosine kinase transmembrane receptor of the ERBB family [50]. Additionally, the main characteristics of basal BC are driven by TGF‐*β* and MYC. More research using BC cell lines and analysis of patient BC datasets points to the significance of this signaling loop in basal BCs but not luminal ones [51]. Therefore, due to the negative expression of targeted molecules like PR, ER, and HER2, the basal‐like BC subtype continues to pose a clinical challenge to mammary oncologists due to the absence of viable therapies. Phosphorylating a particular EZH2 residue, threonine 416, and enhancing EZH′s capacity to complex with SUZ12, cyclin E/CDK2 can improve PRC2 function. PR, ER, ERB2, and BRCA1 were among targeted genes whose expression would be suppressed by local enrichment of the PRC2 complex, which would trimethylate H3K27 surrounding main promoter areas [52]. However, high‐grade mitotic indices, central necrotic or fibrotic zones, and lymphocytic infiltration were characteristics of BLBC. Basal‐like cytokeratin markers like CK5/6, CK14, and CK17 were found in tumors. A number of BLBC also express luminal‐type CKs such as CK8/18 and CK19. BLBC cells exhibit a variety of molecular changes, such as p53 mutations, BRCA1 malfunction, EGFR upregulation, PTEN inactivation, and abnormal production of several noncoding RNA molecules. This is shown in Table 3 [94]. Moreover, TNBC is an aggressive illness with several subgroups, such as basal‐like and claudin‐low, and is characterized by loss of PTEN and numerous deletions or point mutations in TP53. The development of four BCs, including spindle/mesenchymal‐like lesions and PDA, was accelerated by a combination of PTEN loss and p53‐R270H mutant induction [95]. However, basal‐like illness is linked to BC with a BRCA1 mutation. Molecular variations in DNA methylation and gene, protein, and miRNA expression according to BRCA1 status. The average number of mutations, DNA copy number aberrations, and four amplified areas showed significant variations [96]. Additionally, triple‐negative and basal‐like breast tumors frequently have TP53 mutations and have a high degree of genomic instability, while KRAS, NRAS, MET, and AKT1 mutations were found in only one tumor each; PIK3CA gene mutations were found. The most common missense substitution in PIK3CA was a point mutation that changed the amino acid to H1047R [97]. Consequently, BC with BRCA mutations may exhibit unique characteristics that point to the involvement of particular carcinogenic pathways in the development of the tumor. BRCA1‐mutated BC data showed diverse genome‐wide copy number profiles. One of the main causes of this heterogeneity, according to gene expression analysis, is the different numbers of TILs. High TIL counts have a significant impact on genomic profiling of BCs, especially basal‐like and hence BRCA1‐mutated ones [98].

### 2.10. miRNA Signatures and Metastasis in Basal‐Like/Triple‐Negative BC

Recent therapy options for TNBC variations are inadequate due to the disease′s clinical and genetic heterogeneity as well as the absence of clear molecular targets. TNBC with a basal‐like phenotype was exclusively associated with a specific miRNA signature determined by the expression level of miR‐135b. Detections of miR‐135b targets showed a connection of ERBB, Wnt, and TGF‐*β* pathways. This is shown in Table 4 [134], where the possible impact of miR‐126‐3p on the development of basal‐like BC and the tumor microenvironment is shown. Tumor cells of the basal‐like subtype show a decreasing trend in miR‐126‐3p expression when compared to matched adjacent tissues. According to the TMA approach, the expression level of miR‐126‐3p was elevated in the stroma of BCs as opposed to fibroadenomas, and it was lowest in the stroma of basal‐like BCs out of four subtypes [135]. Moreover, breast and other malignancies often have inactivated tumor suppressor PTEN. Molecular categorization, signaling pathways, chromosomal abnormalities, mutational landscape, and decreased expression of miR‐143/145 were all different in transplantable malignancies. Through a mechanism involving induction of RAS signaling, stable knockdown of miR143/145 conferred tumorigenic potential upon poorly transplantable TEN‐deficient tumor cells, increasing their sensitivity to MEK inhibition. Patients with PTEN/miR‐145 loss or PTEN loss/high RAS pathway activity had poor clinical outcomes, and miR‐145 deficit was substantially associated with enhanced RAS pathway activity in basal‐like BC [136]. Additionally, subtype‐specific p53‐dependent alterations include expression of miR‐134, miR‐146a, and miR‐181b. Antagomir KD of miR‐146a was carried out in order to investigate the cellular response to miR‐146a upregulation, which neutralized the effects of p53 loss by increasing apoptosis and decreasing proliferation. The NF‐*κ*B‐dependent extrinsic apoptotic pathway was downregulated, and NF‐*κ*B expression was decreased by miR‐146a upregulation. Antagomir‐mediated miR‐146a KD restored expression of these components, indicating a likely mechanism for miR‐146a‐dependent cellular responses [137]. However, TNBC is characterized by a poor prognosis, rapid progression, and a high risk of metastasis. TNBC cell lines showed a decrease in miR‐126‐3p expression. RGS3 restoration reserved expression of miR‐126‐3p. All things considered, the current work demonstrated that miR‐126‐3p targets RGS3 to function as a tumor suppressor in controlling TNBC cell activity, suggesting that the miR‐126‐3p/RGS3 axis could be a viable therapeutic target [138].

### 2.11. Integrative Clusters

BCs can be categorized into various groups based on their biological, historical, and clinical behavioral characteristics. BC should be categorized according to its underlying biology, which we know is dictated by the somatic genomic landscape of mutations. BCs could be categorized into so‐called intrinsic subtypes, which primarily correlated with HR and HER2 status according to molecular stratification based on gene expression profiling. This is shown in Table 1 and Figure 1 [179]. Moreover, in comparison with deep learning and non‐deep learning–based models, the integrative deep learning model is better. It is demonstrated that HER2‐enriched samples may be categorized into multiple tumor subtypes and identify six subgroups of BC [22]. Additionally, 10 separate subgroups of BC with unique molecular profiles and clinical outcomes are classified using integrative clustering. GEICAM/2006‐14 HER2+ patients were given both TA‐CT and anti‐HER2 treatment. Enrichment of the LP group was observed in residual samples following neoadjuvant ttm as a result of an increase in the integrative cluster and a decrease in the remaining groups [180]. However, mpMRI, which includes DCE MRI, DWT, and T2WI, has transformed the detection and treatment of BC. Clusters linked to notable variations in molecular subtypes, namely, the distribution of luminal A subtype ER and PR, were found in two different patients [181].

### 2.12. Key Driver Mutations in Integrative Clusters

Various subtypes and molecular profiles correlate with verifying outcomes, where BC is a complicated and diverse illness. Total genome sequencing was performed on tumors belonging to the MMTV‐MYC mouse model of BCs microacinar, squamous, and EMT histological subtypes. KRAS activating mutations and EGFR2 amplification are the main causes of KRAS activation signatures downstream of genetic activating events. This is shown in Table 2 [53]. However, individualized BC treatments have been hindered by a lack of trustworthy molecular markers, and the scarcity of fresh tissue is a significant barrier to translational research. The purpose of analyzing gene expression, cDNA‐mediated annealing, selection, extension, and ligation assay was to create DASL, which measured gene expression linked with IHC analysis for ER, PR, and HER2; overexpression of TTF1, LAG‐4, and c‐MYB; underexpression of ABCG2; and overexpression of CXXL9, AREG, and B‐MYB [54]. Moreover, there were no distinct acquired genome characteristics linked to previous anti‐HER2 treatment, and genomic profiles of primary and metastatic HER2+ breast tumors were comparable. According to the biopsy, anti‐HER2 amplified tumors with ERBB2 mutated cases and cases without HER2 changes were categorized by HER2‐driven expression profile [55]. Additionally, cyclin D1 was overexpressed in luminal and normal‐like BCs. CCNE1 was found in significantly higher copy numbers in basal‐like compared to other subtypes; cyclin B1 was overexpressed in poor‐prognosis HR+, luminal B, and basal‐like BCs; and cyclin E1 was a specific marker for triple‐negative and basal‐like tumors [56]. Besides, three gene expression datasets from BCs were analyzed using an integrative bioinformatics method in an effort to identify underlying mechanisms causing resistance to develop and possible treatment approaches to combat these processes. Tamoxifen‐resistant tumors showed increased activity of E2f transcription factors, enhanced expression of cell cycle and proliferation‐related genes, and a strong correlation with the luminal intrinsic subtype. The capacity of phenothiazines to suppress cyclin E2 and stop the growth of tamoxifen‐resistant BC cells confirmed the differences between tamoxifen‐resistant and sensitive tumors [57]. Consequently, three genes with a high frequency of somatic mutations were TP53, PIK3CA, and GATA3. Group A displayed dysregulation of the IGF1R signal and the luminal A‐like subtype. Luminal B was divided into Groups B and C. Amplification of 11q13 CCND1 and activation of the ubiquitin‐mediated proteolysis pathway were characteristics of Group B. Group C displayed reduced expression of ER and PR together with 17q12 [58]. However, there were many subtypes associated with new gene alterations, such as enrichment of certain mutations in GATA3, PIK3CA, and MAP3K1 with the luminal A subtype. Somatic mutations in three genes, TP53, PIK3CA, and GATA3, occurred at > 10% incidence across all BCs. The HER2/p‐HER2/EGFR/p‐EGFR signature within the HER2‐enriched expression subtype was one of the distinct signaling pathways that were found to be dominant in each molecular subtype by integrated analyses. This is shown in Table 3 [99]. Moreover, the TP53 gene was commonly mutated in BC, and mutations have been linked to a poor prognosis. Patients with luminal B, HER2‐enriched, and normal‐like cancers had higher mortality rates due to TP53 mutations, but not those with luminal A or basal‐like tumors [100]. However, one of the most prevalent oncogenic drivers of all cancer types is the loss of the PTEN tumor suppressor. The primary negative regulator of PI3K signaling is PTEN. PI3K*β* inactivation boosted production of immune stimulatory molecules in the PTEN null context and decreased STAT3 signaling, which in turn enhanced antitumor immune responses [101]. Furthermore, the proposal is supported by established associations for BRCA1/2 and the apolipoprotein B editing complex. Three localized deletions, one amplification, and mutations in ARAP3 were found for BRCA1/2.3 kinase KIF13A, KIF1B, and KIF4A; three ubiquitins USP45, UBR4, and UBR1; two demethylases KDM5B and KDM5C; and other genes linked to DNA damage pathways were among 50 altered genes for two APOBEC signatures that were found [102].

### 2.13. miRNA Signatures and Metastasis in Integrative Clusters

A lot of interest has been focused on circulating miRNA as potentially useful new biomarkers for BC. Plasma levels of miRNAs such as miR‐148b, miR‐133a, and miR‐409‐3P were significantly higher in cases of BC than in healthy controls. Scientists discovered that BC cell lines secreted both miR‐148b and miR‐133a throughout the in vitro testing phase, indicating their potential as secretors and potential tumor origin. This is shown in Table 4 [139]. Moreover, BC growth and progression are significantly influenced by miRNAs. It was shown that Has‐miR‐127 is an independent predictor of BC. BC tissues and cell lines showed downregulated expression of bmiR‐127, while overexpression reduced BC cell invasion, migration, and proliferation, and miR‐127 knockdown significantly boosted these processes [140]. Furthermore, a set of sporadic and familial breast tumors was subjected to miRNA profiling, which revealed a substantial correlation between miRNA‐342 and ER levels. The overexpression of miR‐342 decreased ID4 and raised BRCA1 expression, suggesting that this pathway may play a part in BC. Since the majority of ER‐negative cases were BRCA1 mutant, we hypothesized that the mechanism we showed may contribute to the decreased BRCA1 expression commonly seen in non‐BRCA1 mutant BCs and may be a contributing factor in some familial cases that fall into diverse categories of non‐BRCA1 or BRCA2 mutant cases [141]. However, AGR2 and miR‐135‐5p expression levels were examined in various cell lines of BC. BC cells′ AGR2 expression was negatively controlled by miR‐135‐5p, which increased their doxorubicin sensitivity. In vivo BC cells′ doxorubicin sensitivity was elevated when miR‐135b‐5p was upregulated [142].

### 2.14. Special Histological Subtypes

Different clinical and morphological characteristics in certain situations from more prevalent invasive carcinoma are categorized as special histological subtypes of BC. Although the histological characteristics of ILC and NST differ, there is debate regarding the impact of histological subtypes on survival. The nodal stage was found to have a differential impact on survival. ILC patients with pN0/pN1 tumors had a higher survival rate than NST patients, whereas ILC patients with pN2/pN3 tumors had a lower survival rate. This is shown in Table 1 and Figure 1 [182]. Moreover, another research study conducted found that a special histological subtype of cancer is thought to have a favorable prognosis. All special subtypes had mean RSs that were lower than those of IDC patients. The proportion of patients with high RS results rose from 9.5% to 16.8% when the high RS threshold was lowered from 31 to 25 [23]. Consequently, more than 25% of all invasive breast tumors are histologically different kinds, which have unique morphological characteristics. The pattern of gene copy number aberrations discriminated with ER status and histological grade according to hierarchical clustering, and samples from each of the histologically distinct kinds of BC clustered together preferentially. One hundred and forty‐five transcripts were found to be significantly overexpressed when amplified in histologically distinct types of BC through the use of integrative aCGH and gene expression analysis [24]. Furthermore, diagnostic guidelines were cautious regarding predictive power based on histological subtype alone, and whether they are in their pure form or in conjunction with other histological subtypes is yet unknown. The cribriform subtype had the best prognosis when pure histological subtypes were pooled with tumor stage and age in a linear random effects model, but male BC had the worst outcome [183]. In addition, about 25% of invasive BCs are categorized as histological subtypes, whereas the majority are classed as invasive carcinoma not otherwise described, while some histological special types, such as micropapillary carcinoma, are distinct entities. Hierarchical clustering research also showed that other types, like tubular and lobular carcinoma, are highly similar at the transcriptome level. Histological special‐type cancers, with the exception of apocrine, are homogeneous and only belong to one molecular subtype, whereas IDC, NOS, and ILC, when categorized by expression profiling, encompass all molecular BC types [184]. Therefore, searching for new markers for TNBC has been the focus of a lot of research, but there is little data on the relationship between histological characteristics and survival in TNBC. The most common histological subtypes were lobular carcinoma (3.4%), metaplastic carcinoma (4.4%), and NST (88.4%) [185].

### 2.15. Key Driver Mutations in Special Histological Subtypes

Accuracy and interlaboratory reproducibility of assays available for measuring overexpression of HER2 in BC have demonstrated significant issues. Grade 1 NST carcinoma, which only 1.4% have been demonstrated to overexpress HER2, as well as infiltrating lobular and peculiar forms of carcinomas, might not require regular testing at presentation. This is shown in Table 2 [59]. However, NAC was an accepted therapeutic technique for BC in order to downstage the disease. IDC can be effectively treated using recognized regimens. Patients receive breast conservative therapy less frequently than IDC patients, and their clinicopathological response to NAC was noticeably worse [60]. Moreover, there have been conflicting reports about the incidence of cyclin D1 overexpression and its relationship to CCND1 amplification and the prognosis of patients with BC when clumps of big gene copies were seen. In 14.5% of cases, there was strong cyclin D1 expression and CCND1 amplification. ER and PR were positively correlated in cyclin D1, and both cyclin D1 overexpression and CCND1 amplification were found to be inversely correlated with the immunohistochemistry panel of basal‐like markers [61]. Therefore, c‐myc protein is thought to promote expression of HSP7O by acting on the HSP7O promoter. The majority of heat shock protein, HSP7O, shields cells from a range of stressful stimuli. In malignant cells from 37 carcinomas (63%), the expression of c‐myc protein and HSP7O were similar. Five carcinomas had HSP7O as the predominant protein, while 17 carcinomas had c‐myc protein expression over HSP7O [62]. However, the effectiveness of chemotherapy in avoiding recurrences and prospectively assessing the prevalence of HRFs in patients with unilateral retinoblastoma undergoing enucleation. Fifty‐three patients had postlaminar optic nerve involvement, 42 had significant posterior uveal invasion, 15 had localized choroidal contemporaneous with lamina or prelamina optic nerve involvement, and 15 had concurrent peripapillary or large choroid and past laminar optic nerve involvement. This is shown in Table 3 [103]. Consequently, the fact that BRCA2 and RB are located on the same chromosome arm suggests that such allelic losses may have a significant impact on one or both of these genes. The more distal of the two segments, which contained the RB gene, was situated in a 9‐cm interval flanked by marker loci D13S328 and D13S172, while the more proximal segment, which contained the BRCA2 gene, was situated in a 6‐cm interval flanked by marker loci D13S289 and D13S267. Tumors of solid tubular histologic type had a higher frequency of allele loss on 13q (55%) compared to other kinds (36%) [104]. Therefore, p53 connection with HER2/new HER expression, tumor histology, prognosis, and epidemiologic risk factors are four facts of node‐negative BC. Compared to lobular (9%) and ductal (23%), MEDs had a much greater prevalence of p53 expression, which was 68%. p53 was detected in a small percentage of mucinous and absent tubular and papillary low‐grade carcinomas [105]. However, TP53 hotspot mutation was examined in a variety of p53 immunophenotypes. There were 176 out of 288 cases (61%) with extreme expressiveness of any kind. Extreme IHC phenotypes were associated with six substitution exon mutations that were found [106].

### 2.16. miRNA Signatures and Metastasis in Special Histological Subtypes

miRNAs used in BC as biomarkers, such as RNU6B, let7a, miR‐17, miR‐27b, miR‐125a, miR‐125b, and miR‐206, were examined pairwise to identify pairings that had the strongest correlations with histological categories of 27 BC samples. HER2‐negative and HER2+ groups were distinguished by a single miRNA pair. Two miRNAs were chosen: miR‐125a/miR‐206 and miR‐125a/miR‐125b, which were luminal, HER2+, and basal. This is shown in Table 4 [143]. Moreover, miR‐218 has multiple functions in different stages in BC. The expression profile of miR‐218 and the B‐cell‐specific BMI1 gene, one of the putative targets of miR‐218 in 33 paired breast tumors and their surrounding normal tissues, was compared to the clinicopathological characteristics of patients [144]. Therefore, patients with ER‐positive BC are linked to miR‐143. Patients with ER‐positive BC who were in the miR‐143 high expression group had better OS due to increased infiltration of anticancer immune cells, decreased procancer immune cells, and enrichment of genes related to T helper cells [145]. Consequently, in patients with locally advanced TNBC, miR‐143 could distinguish between a pathological complete response and no polymerase chain reaction. Low miR‐143 and high levels of GSK3‐*β*, RAF1, and p21CIP1 expression were linked to BC. miR‐143 also reduced protein levels and phosphorylation status of multiple oncoproteins involved in AKT, Wnt/*β*‐catenin, SAPK/JNK, FAK, and JAK/STAT signaling pathways [146]. Besides, therapy resistance is a major contributor to cancer mortality, yet molecular mechanisms remain incompletely understood. miR34a, a key tumor suppressor downregulated in BC stem cells, enhances therapeutic sensitivity by targeting HDAC1 and HDAC7, thereby preventing HSP7O K246 deacetylation and promoting autophagic cell death. The miR‐34a‐HDAC1/HDAC7‐HSP7O axis represents a promising biomarker and therapeutic target for overcoming resistance in BC [147].

### 2.17. Invasive Lobular Carcinoma

Ten percent to 15% of all cases of BC are ILC, which is the second most common histologic subtype of disease after IDC. IDC and ILC are different entities, as evidenced by reports of multiple studies conducted over the last 5 years with the goal of comprehending ILC at clinical, cellular, and molecular levels. This is shown in Table 1 and Figure 1 [25]. However, about 10% of BC are ILC, which seem to have a unique biology. ILC metastasized to the ovaries and gastrointestinal system more frequently than IDC. Incidence of contralateral BC was greater for ILC patients than for IDC patients [186]. Moreover, up to 15% of all occurrences of BC are ILC, and the most prevalent special morphological subtype of disease is HR‐positive, HER2, and p53 and basal marker negative, low histological grade, low mitotic index, and typically satisfactory response to endocrine treatment [187]. Therefore, IDC‐L′s clinicopathologic characteristics and prognosis are unclear, and it is now acknowledged as a separate subtype of BC. IDC‐L was less likely to overexpress ER‐2/neu, had a lower histologic grade, and tested positive for ER and PR than IDC. IDC‐L showed a worse 5‐year disease‐free survival than IDC and a greater incidence of nodal metastases in spite of these positive prognostic characteristics [188]. Consequently, another research says that ILC is the most prevalent of BC. Multivariate analysis comparing patients with classical ILC to those with solid and mixed nonclassical ILC. A multivariate study compares individuals with luminal A ILC to those with luminal B, HER2‐positive, and triple‐negative subtypes [189]. Besides, after IDC, ILC is the second most prevalent histological type. Comparison with IDC (16.2%) and ILC (0%) showed a significantly lower HER2+ rate. Patients with ER+/PR+/HER2− subtype and median survival time following recurrence were 4.2 and 5.6 years, while mean DFS for ILC and IDC patients was 2.9 and 3.1 years [26].

### 2.18. Key Driver Mutations in Invasive Lobular Carcinoma

IBC with a sizable in situ component was analyzed using CGH and FISH; only the invasive component showed high‐level c‐MYC amplification. Overexpression of TERT and FBL, two c‐MYC target genes. This is shown in Table 2 [63]. However, the transcriptional regulator MYC gene, also known as c‐MYC, is closely linked to both cell differentiation and proliferation. MYC amplification was seen in two patients (13%), although only in designated invasive carcinoma zones. Only ductal in situ and invasive zones were found to have polysomy of chromosome 8 (33%), which was observed in five patients [64]. Therefore, another article published that about 10% of human breast tumors are invasive lobular carcinomas. Overexpression of cyclin D1 protein in invasive lobular cancer elevated protein levels related to cell cycle regulation [65]. Consequently, two kinds of lobular tumors promote methylation of five cancer‐related genes, such as RASSF1A, HIN‐1, RAR‐*β*, and cyclin D2, and twist was assessed regarding RASSF1A, HIN‐1, RAR‐*β*, and cyclin D2 genes, and methylation patterns of lobular and ductal carcinomas were comparable, which indicates that promoter hypermethylation patterns of lobular and ductal carcinomas were comparable, so this indicates that promoter hypermethylation‐induced gene silence is probably significant in both disease types [66]. Besides, scientists recruit 209 and 109 primary BC patients with a diagnosis of IDC, ILC, or IDC‐L in order to examine clinicopathological features and prognosis of mixed invasive ductal and lobular carcinoma. The survival benefit of IDC‐L vanished when clinicopathological variables were taken into account. IDC‐L had greater HRs than IDC in Grade 1, Grade 2, ER‐positive, and ER‐negative categories according to subgroup analysis [67]. However, CDH1 germline mutations exclusively predispose to ILC due to genetic susceptibility. BRCA1 was linked to a reduced ILC fraction, and BRCA2 and TP53 in wild‐type patients. This is shown in Table 2 [107], where we found interesting topics that about 10% of IBC are ILC, whereas the majority are ductal carcinomas. Genetic susceptibility to BC is suspected in clinical practice of four genes: BRCA1, BRCA2, TP53, and CDH1. Primary susceptibility gene for diffuse gastric cancer, but the CSH1 family had a high number of lobular breast tumors. Researchers also found that it was a susceptibility gene for ILC [108]. Therefore, the authors said that 5%–15% of IBCs are caused by ILC. A higher pathological full response rate and a larger reaction to NACT were seen in atypical ILCs [109]. Consequently, one subtype of aggressive BC that does not respond well to treatment is ILC. Promoting cell survival and quick development of invasive mammary tumors that mimic histological and molecular characteristics. ER status, growth kinetics, metastatic behavior and tumor microenvironment of CLC, and loss of E‐cadherin caused cell dissemination and apoptosis [110].

### 2.19. miRNA Signatures and Metastasis in Invasive Lobular Carcinoma

According to Saber (2017), miR34a is used as an invasive indicator for BC cells, and 13 studies from nine publications, including 1858 BC cases and 494 controls, were analyzed. The pooled sensitivity and specificity of miR34a were 85.5% and 70.0%, respectively, with an AUC of 0.80, indicating good diagnostic performance. Subgroup analyses showed higher accuracy in tissue‐based samples of invasive BC among Caucasian populations. This is shown in Table 4 [148]. However, single‐gene housekeeping candidates miR‐16‐5p, miR‐29a‐3p, and miR‐222‐3p are appropriate. Strong expression of 29 human miRNAs was observed, and 21 of these miRNAs, such as miR‐16‐5p and miR‐29a‐3p, showed stable expression. miR‐29a‐3p and miR‐16‐5p were excellent housekeeping candidates [149]. Furthermore, comparison between the GLM and the control group in BC had significantly greater serum expression of miR‐451a and miR‐5571‐3p. Comparison with the control group GLM showed substantially greater expression of miR‐451a and miR‐5571‐3p, where GLM increased levels of CLN6 [150]. Consequently, 50 women with Stage I–II BC under 45 years of age were treated. PCR examination was used to assess expression of miR‐145, ‐182, ‐21, ‐27a, ‐29b, and ‐34a in tumor tissue. Tumor stage of BC found lower levels of miR‐27a and higher expression of miR‐182, ‐21, and ‐29b [151]. However, metastatic relapse quickly results in multiorgan failure, which is typically the cause of mortality linked to BC. Significant associations with advanced disease were found for miR‐146a‐5p, miR‐126‐5p, miR‐122‐5p, miR‐16‐5p, miR‐142‐3p, miR‐223‐3p, miR‐103a‐3p, miR‐221‐3p, miR‐21‐5p, and miR‐30d‐5p. Higher amounts of miR‐223‐3p, miR‐146a‐5p, and miR‐148b‐3p were seen in tumor histotypes that were ductal as opposed to lobular [152]. Besides, we have found interesting findings that miR‐21, miR‐155, miR‐27a, miR‐205, miR‐145, and miR‐320a are linked to breast tumors. The functions of these miRNAs in relation to resistance to treatment with several anticancer agents, including therapies such as trastuzumab and tamoxifen, were investigated [153].

### 2.20. Tubular Carcinoma

Retrospective pathologic examination of 636 breast carcinomas from 611 individuals identified 19 tumors with characteristics combining low‐grade T&D and 12 tumors that were pure low‐grade TC. A 15‐year life table study revealed that survival rates for TC, T&D, and controls were 100%, 72%, and 33%, respectively. In comparison with controls (67%), axillary metastases were seen in 8% of TC and 21% of T&D. This is shown in Table 1 and Figure 1 [190]. Moreover, TC is known to have a good prognosis, but it is still unclear whether this subtype of BC behaves like other low‐grade luminal A‐type cancers or if it is separate from BC. Longer disease‐free and BC‐specific survival were linked to TC [191]. However, TC was used to determine whether the separation of IDC with a major MTC, which has a tubular component of 50%–75%, from the mixed type of TC, which has a tubular component of more than 75%, was successful. Four groups were monitored, and 10 of 20 patients with TC passed away during this time compared to 37 of 40 controls. To conclude, 29 of 320 controls and 10 of 16 patients with ductal carcinoma MTC passed away [27]. Furthermore, evaluate the survival and axillary lymph node involvement rate for mucinous and tubular BC, which are thought to have a very excellent prognosis. Then, for 71.2% of patients, SLNB was the only method used to determine axillary lymph node status, and TC and MC ALNI rates were 17.9% and 18% [28]. Consequently, BTC has been thought to have a relatively good prognosis and is a known histologic variation of infiltration of ductal carcinoma. Compared with Grade I IDC patients, TCB patients had a higher prevalence of low‐risk tumors, and low‐risk Grade I IDC patients′ incidence of nodal metastases at presentation was much reduced in low‐risk TCB cases [192].

### 2.21. Key Driver Mutations in Tubular Carcinoma

BCs′ ER, PR status, and HER2 gene amplification were well known. In order to determine the prevalence of HER2 amplification for tubular carcinomas, scientists examined 55 cases of BTC patients, five of whom had metastases to axillary nodes. The prevalence of invasive ductal carcinoma with HER2 gene amplification was substantially lower. This is shown in Table 2 [68]. Consequently, HER2 gene amplification for TC and another scientist evaluated the frequency of gene amplification from 55 BTC, and HER2 gene amplification was detected from the tumor analyzed, and the majority were ER‐positive [69]. Therefore, despite the existence of p53‐independent regulatory mechanisms, p21 is also associated with cell differentiation and is controlled by wild‐type p53. Preinvasive lesions also showed p21 expression, while normal ducts were either focally and faintly positive or negative. p21 expression was considerably less common in lobular carcinomas and linked to poor tubule formation and high histological grade. Increased proliferation was associated with p21‐positive, but this appeared to vary with histologic grade [70]. Besides, cyclin E is a G cyclin that is necessary for the cell cycle transition from G1 to S phase. Cyclin E has been studied in both malignant and nonmalignant BCs. More than 5% of reactive cells were seen in 28% of IC [71]. However, up to 40% of TNBCs have genetic changes in RB, and these changes are common in tumors that have neuroendocrine development. Only BCs with biallelic RB1 deletion showed a decrease in Rb protein expression, showing a reduction of expression of Rb and biallelic genetic inactivation of RB1. TP53 (53%) and PIK3CA (45%) genes were the most altered genes. This is shown in Table 3 [111]. However, one of the most often altered human tumor suppressor genes is phosphatase and tensin homolog deleted on chromosome ten. PTEN in 10 healthy people and 43 patients with BC. The observed heterozygosity is lower than the anticipated heterozygosity in benign and malignant breast disease, according to microsatellite analysis at the PTEN locus utilizing D10S215, D10S541, and D10S579 markers [112]. Moreover, IDC with poor differentiation that often resembles normal or atypical medullary carcinomas is known as BRCA1‐associated carcinomas. Compared to sporadic age‐matched controls, BRCA2‐associated breast carcinomas atypically have a higher grade. BRCA1 cancers are more likely to be p53‐positive and ER− and PR−, where P‐cadherin and/or cytokeratin 5/6 are examples of basal markers that are expressed in BRCA1 breast carcinomas [113].

### 2.22. miRNA Signatures and Metastasis in Tubular Carcinoma

Breast carcinogenesis is a multistep process that is characterized by genetic and epigenetic alterations. High TNM stage left‐sided tumor, metastasis, high‐grade illness, higher axillary nodal involvement, lack of ER and PR, and low antigen Ki67 expression are all associated with miRNA‐127 expression. This is shown in Table 4 [154]. However, another research has been conducted. Those downstream proteins in HUVECs treated with Exo‐SKF or Exo‐STIMI1‐KO were significantly suppressed, including AKT/mTOR, Raf/ERK, and p38 expression [155]. Moreover, two morphogenetic mechanisms linked to tumor invasion and metastasis are angiogenesis and exosomes. miR‐218 mimic was effectively transported to MDA‐MB‐231 cells via ADMSC exosomes. In MDA‐MB‐231 cells, exposure to exosomes carrying miR‐218 markedly reduced the expression of miR‐218 target genes such as Runx2 and Rictor [156]. Therefore, it had been demonstrated that mesenchymal stem cell–derived exosomes containing miRNAs control the biological activity behavior of BC tumor cells, which was inhibited by miR‐342‐3p. It was established that miR‐342‐3p and ID4 had a binding site. Effects of miR‐342‐3p on chemoresistance may be reversed by ID4 [157].

### 2.23. Mucinous Carcinoma

The prognosis for MBC is generally favorable, and a total of 111 individuals with MBC were found. Seventy‐one patients, about 64%, had radiation and a lumpectomy. Node positivity was linked to higher tumor size, and the mean tumor size for node‐positive patients was 2.7 cm, where node‐positive patients was 1.5 cm, and 14 patients, about 13% of patients, developed lymph node metastases. This is shown in Table 1 and Figure 1 [29]. Moreover, morphological and clinicopathological characteristics and prognoses of 175 women with MBC were examined. They were separated into two types: mixed and unmixed, where the unmixed type produces more extracellular mucus and has less frequent nodal involvement than the mixed kind [193]. However, another research has been conducted that all mucinous carcinomas had MUC2 expression, where only 11.1% of IDC and none of the invasive lobular and medullary carcinomas did, while medullary carcinomas do not express MUC1, and IBC does [194]. Therefore, one uncommon histologic form of mammary neoplasm is pure MBC, and 38% were moderately differentiated, 53% were well differentiated, and the remaining 9% were either anaplastic or poorly differentiated. Then, 56% of cancers were distributed fairly equally across upper inner, lower inner, lower outer, and central quadrants, with 44% of tumors found in the upper outer quadrant. Then, 12% had regional nodal involvement, 2% had distant metastases, and 86% had only localized disease at the time of surgery without nodal or distant illness [195]. Consequently, there is little evidence to support the efficacy of breast‐conserving treatment for mucinous carcinoma. Then, 11% of pure mucinous carcinomas had comedo‐type EIS. PMC had lower rates of lymphatic vessel invasion (4%) and nodal involvement (4%) compared with mixed carcinoma [196]. Besides, scientists said that up to 2% of BCs are mucinous carcinoma, an uncommon entity that has been found to have a different gene expression profile from IDC of no special type, IDC‐NSTs. Using antibody treatment with ER, PR, HER2, Ki67, cyclin D1, cortactin, BCL‐2, p53, E‐cadherin, basal markers, neuroendocrine markers, and ET1, 35 pure and 11 mixed MBC were evaluated [197].

### 2.24. Key Driver Mutations in Mucinous Carcinoma

One of the most uncommon BCs that has a good prognosis is MC. DFS and DMFS were noticeably poorer in HER2+ patients and HR‐positive/node‐negative patients with tumors larger than 3 cm. This is shown in Table 2 [72]. However, HER2 is rarely overexpressed or amplified in pure MBC, whereas the OS rate was considerably worse for HER2+ PMBCs than for HER2‐negative PMBCs. Higher TNM stage, nuclear grade, and nodal metastasis were found to be associated with a worse OS rate for patients with PMBCs [73]. Moreover, 38 patients had 32% carcinoma DNAs, where the c‐MYC proto‐oncogene seemed to be amplified two to 15 times in five PBC, and a nongermline c‐myc‐related fragment of verifying size was found. All tumors with a genetic change in the c‐myc locus were invasive ductal carcinomas, with three exceptions [74]. Furthermore, a diverse set of aggressive initial breast tumors that can differentiate into sarcomatous and carcinomatous elements is represented by MBC, where MYH11 and amplification of ERCC5 and FGF14 and next‐generation sequencing identified driver mutations HRAS and PIK3R1 that may have contributed to the tumor phenotype [75]. However, the most interesting fact is that PMBC is known as MC with micropapillary features that exhibit micropapillary characteristics. Recurrent 6q14.11‐q27 losses, 8p11.21‐q24.3 gains, and common mutations affecting TTN, GATA3, SF3B1, and TP53 were all present in MPMCs and PMCs, which also showed a low mutation load, while PIK3CA mutations were only found in PMCs; GATA3, TP53, and SF3B1 mutations were often seen in MPMCs. This is shown in Table 3 [114]. However, growing knowledge in diverse biology has grown, which must be thanks to gene expression profiling, which also holds promise for improving clinical treatment. Some predictors, like tumor size, gene expression‐based categorization, and TP53 mutant status, while highly proliferating luminal cases developed disease more slowly and had the highest mortality after 5–8 years, and basal‐like and ERBB2+ gene expression subgroups had very high mortality during the first 2 years [115]. Therefore, multiple tumors appeared in patients′ lungs, bones, and lymph nodes within 6.5 years after the oophorectomy, and histology findings verified that the tumor was mucinous adenocarcinoma. Multigene panel testing revealed germline BRCA2 variations in addition to substantial tumor mutation load and microsatellite instability in mucinous adenocarcinoma lacking MSH2 and MSH6 expression [116]. Consequently, one of the most prevalent genetic abnormalities in IBC is activating mutations in PIK3CA. PIK3CA mutations in an undifferentiated comparison sample of IDC. ADH 2/3, DH 2/3, and columnar cell change 1/5 were found to have PIK3CA hotspot point mutations, which is interesting because it suggests that PIK3CA mutations may be involved in the proliferation of breast epithelial cells [117].

### 2.25. miRNA Signatures and Metastasis in Mucinous Carcinoma

miR‐217 expression was high in BC tissues, advanced tumor stage, TNBC, and high histological grade. Dual luciferase reporter assay results showed that miR‐217 directly targets and suppresses DACH1′s transcriptional activity. This is shown in Table 4 [158]. Therefore, another similar research investigated that PMBC is a rare histological form of BC that is distinguished by high mucin formation. Downregulation of miR‐143 and miR‐224‐5p in mucinous carcinoma tissue was demonstrated in PMBC with lower cell proliferation, lower HER2+, and higher HR‐positive compared with IDC‐NSTs [159]. Consequently, in TNBC and normal samples, lincRNA‐ROR was elevated. TNBC cell lines demonstrated that abnormal lincRNA‐ROR expression increases invasion and metastasis, and siRNA loss of function reverses this process. LincRNA‐ROR is a competitive endogenous ceRNA that increases the expression of MUC1 by sponging miR‐145, which affects the location of the E‐cadherin membrane [160].

### 2.26. Adenoid Cystic Carcinoma

Less than 0.1% of BCs are ACCs, which are uncommon tumors. Uncertainty surrounds its cellular genesis in the breast. Histologically, ACC of the breast has similarities with ACC of salivary glands. This is shown in Table 1 and Figure 1 [30]. Moreover, ACCs are malignant tumors that seldom develop in the breast. They often develop in the bronchi and salivary glands. Atypical growth with a cribriform growth pattern and mucosal fluid surrounding tumor nests and within tumor ducts was found during pathological analysis of VAB tissue [198]. Therefore, out of 40,350 IBC cases, 37 instances of ACC of the breast were found, and out of 27 surgical pathology slides that were available for inspection, only 14 had histological confirmation of ACC [199]. Consequently, in bigger lesions, ACC displayed a range of enhancement kinetics from washout kinetics to sustained enhancement. The tumor had enthusiastic radiotracer uptake on molecular breast imaging, although PET activity was not always present [200].

### 2.27. Key Driver Mutations in Adenoid Cystic Carcinoma

Eight patients (38%) had eight cases (38%), and eleven cases (52%) had tumors that were positive for NICD1 expression, c‐myc expression, and p63 expression. Treatment with a mostly solid pattern that included orbital exenteration, local recurrence, and death was linked to positive NICD1 expression. Death, distant metastases, local recurrence, and a mostly solid pattern were all linked to negative p63 expression. Perineural invasion was linked to a greater proportion of tumor cells that stained for c‐MYC. This is shown in Table 2 [76]. However, adenomyoepitheliomas are thought to be benign or to have little chance of becoming cancerous. In basal‐like breast tumors, unchecked proliferation and cellular immortalization are caused by MYC dysregulation [77]. Besides, two distinct tumor clones featuring concordant HRAS and heterogeneous ARID1A mutations will define salivary gland EMC. The latter appeared mostly in solid oncocytic differentiation and appears to be a second hit, indicating a possible morphomolecular link [78]. Therefore, in 18 of 19 PAC, Er*α*36 expression was shown to be mostly cytoplasmic and membrane‐based, where Er*α*36‐positive expression was consistently expressed by 100% of cells in PAC. All three conventional steroid receptors were consistently negative in all ACC; however, Er*α*36 was found in eight of 11 cases and exhibited a subcellular pattern of expression that was comparable to that of APC [79]. However, renal metastases were associated with increased PTEN promoter methylation and decreased levels of normal PTEN transcript in rarely ACC spread. PI3K/AKT pathways coexisting with mutations and epigenetic activations might be the cause of ACCs′ typically aggressive progression. This is shown in Table 3 [118]. However, 33 breast ACCs were 16 conventional ACCs and 17 solid variation ACCs, and similar to the more prevalent form of ACC, solid ACCs showed MYB protein overexpression and basaloid shape with exclusive or major epithelial cell population linked to impaired myoepithelial differentiation [119]. Furthermore, a number of benign and malignant neoplasms believed to be connected to AGMLGs include BRCA1, BRCA2, and PIK3CA mutations. These markers include three cases of mammary‐type carcinoma, five cases of hidradenoma papilliferum, and nine cases of primary extramammary Paget disease [120].

### 2.28. miRNA Signatures and Metastasis in Adenoid Cystic Carcinoma

DNA methylation in TNBC gene silencing and creation of DNA methylation inhibitors to activate TSGs that have been silenced. Gene expression is largely controlled by histone modifications, namely, histone acetylation and deacetylation. A possible approach to individualized treatment plans is a combination of DNA methylation inhibitors, HDACi, and miRNA‐based treatments. This is shown in Table 4 [161]. Consequently, miRNA signatures and suppression of Er*α* expression/Er*α* signaling altered the expression of several pro‐oncogenic and tumor suppressor proteins, and genotoxic effects were caused by oxidative estrogen metabolites. TNBC is a type of aggressive BC that lacks expression of ER, PR, and HER2 [162]. Therefore, miR‐27b and miR‐27b as tumor suppressors are upstream factors that control the stability of VDR mRNA. Expression of miR‐125a and miR‐27b regulates VDR, which is upregulated in malignant breast tissues relative to nearby normal tissues [163]. Besides, NPC cells experienced cell cycle arrest at the M phase as a result of downregulating c‐MYB expression, which also prevented cell growth. c‐MYB directly binds to the promoter of miR‐143, transactivating it [164]. Additionally, by altering different signaling pathways and biological processes, miRNAs, which are post‐transcriptional regulators of gene expression, control target genes [165].

### 2.29. Secretory Carcinoma

IBC and SBC are uncommon histological subtypes that are thought to exhibit a slow clinical course. Mean tumor size was 3.5 cm, with a range of 0.3–6.8 cm. Positive involvement of axillary lymph nodes was seen in eight individuals (15.4%). TNBC made up the majority of molecular categorization (65.4%). This is shown in Table 1 and Figure 1 [31]. Moreover, palpable lumps, bloody nipple discharge, and screening‐detected anomalies were clinical symptoms. Breast sonograms revealed round, oval, or tubular masses with hypoechoic or isoechoic internal echo textures and relatively well‐circumscribed or partially microlobulated margins. Except for a 3.5 cm mass with axillary lymph node metastases, the majority of lesions were single nodules or clusters of nodules that were 1 cm or less [201]. However, using integrated diagnostic approaches such as morphology, immunohistochemistry, and molecular pathology, about 19 patients were first identified as SC. Potential indicators in 445 breast tumors were verified. Nine SCs, three ACCAs, three CHCs, three IDCs, and one microglandular adenosis among 19 previously identified SCs were reclassified [202]. Moreover, metastatic deposits in four of 11 patients who had axillary nodes removed displayed the same secretory characteristics as the original tumor. One of four patients, a female patient, passed away from a disseminated tumor after 10 months, because children and teenagers are not the only ones that have these particular patterns of cancer [203]. Additionally, another uncommon kind of BC that has an indolent behavior is SBC. Basal‐like marker CK5/6 or EGFR has an 87% expression rate, and 13 instances (87%) had a basal‐like phenotype. So, SBC expresses basal‐like markers, making it a unique subtype of IBC [204].

### 2.30. Key Driver Mutations in Secretory Carcinoma

HER2 expression levels in normal and HER2‐overexpressing breast carcinomas differ, and it has been shown that HER2 plays a crucial role in the development of tumors [80]. However, CAFs, among the most significant constituents of the tumor matrix, are constantly active by blocking Wnt/*β*‐catenin pathways with neutralizing antibodies against CTHRC1 or inhibitor Dickkopf‐1; CAF‐induced malignant phenotypes of BC cells were considerably reduced. This is shown in Table 2 [81]. Moreover, seven out of 11 normal BC and every breast carcinoma had c‐MYC product. It was shown that the c‐ERB2 protein is present in 45% of IBC and 29% of NDC [82]. Consequently, another research has been conducted that cyclin E1 is one of the promising biomarkers in ER+ BC. Patients may be categorized as prospective responders or nonresponders based on cyclin R1 expression. Cyclin E1 and cyclin E2 are closely linked so that both estrogen target genes can promote antiestrogen resistance and have elevated expression in BC [205]. However, ETV6‐NTRK3 translocation and PDGFRB mutation were found by next‐generation sequencing of 211 cancer‐related genes. BRCA1‐2, TP53, RAD51C, and RAD51D mutations are the most frequently changed molecules in male aggressive BC. This is shown in Table 3 [121]. Therefore, Pyk2, SAPK/JNK, phosphatase, and tensin homolog were shown to be more abundant in the serum of cancer patients. AA cancer serum has significantly higher levels of c‐kit and RB than CA cancer serum [122]. However, IGF 1Ec expression was measured in 30 instances with normal endometrium, in 30 cases with endometrial hyperplasia, and in nine cases of non‐neoplastic endometrial tissue to tumor. The relationship with IGF 1Ec and p53, surviving, phosphatase, and PTEN was elevated as a relationship between combined expression and clinicopathological factors [123].

### 2.31. miRNA Signatures and Metastasis in Secretory Carcinoma

Exosomes containing miR‐127‐3p were separated and eliminated extracellularly. Protein–protein interaction analysis revealed MYCN to be the most significant hub for RAB27A‐OE RCC cells, and the ceRNA network showed that MAPK4 and miR‐127‐3p have a significant interaction. This is shown in Table 4 and Figure 1 [166]. Moreover, patients with BC who had LNM and a low differentiation degree had greater levels of miR‐520g expression, while patients with BC who had mammary gland invasion and poor p53 expression had significantly higher levels of miR‐520g expression [167]. However, miR‐520/373 members were overexpressed in MDA‐MB‐231 cells, and TGF‐*β* signaling was downregulated, according to mRNA profiling. Since the effects of miR‐520/373 overexpression on suppression of Smad‐dependent expression of metastasis‐promoting genes, parathyroid‐related protein, plasminogen activator inhibitor‐1, and angiopoietin‐like 4, as well as tumor cell invasion, were replicated by silencing TGFBR2, the metastasis‐suppressive role of miR‐520/373 can be described as direct suppression of TGFBR2 [168]. Lastly, scientists found that comparing treated BC MCF7 cells with control cells, miR‐126 was overexpressed. Expression of miR‐126 has been linked to MCF7 cells being halted at the G1 phase and a reduction in cell growth [169].

### 2.32. Medullary Like Carcinoma

MCs have a better prognosis than Grade 3 mammary carcinomas. Biological characteristics similar to those of basal carcinomas are linked to poor prognosis. Then, 36% of carcinomas had a fibrotic center, whereas medullary carcinomas presented 3% of tumors with a fibrotic focus. This is shown in Table 1 [32]. However, MBCs are characterized by intense chronic inflammation and high‐grade and restricted morphology. Basal phenotype is linked to them and triple positivity for basal markers CK14, EGFR, and 34*β*E12, which included patients with a markedly lower DFS rate over 10–15 years, and it was substantially associated with ER negativity [206]. Consequently, MBC is a pathogenic subtype that is an uncommon and challenging pathogenic subtype to diagnose. P‐cadherin, MIB1, ERBB negativity, and p53 positivity were found in MBC [207]. Besides, the most recent research has identified MBC as belonging to the spectrum of basal‐like carcinomas, which is consistent with TP53 mutations identified in MBCs. Higher rates of KRT 5/6 expression and higher rates of gains and losses compared to BLCs; recurrent 10p, 9p, and 16q gains; 4p losses; and 1q, 8p, 10p, and 12p amplicons distinguish MBCs as a unique entity within the basal‐like spectrum [208].

### 2.33. Key Driver Mutations in Medullary Like Carcinoma

IBC with medullary characteristics represents a distant BC phenotype, and the importance of CK5/6 and/or EGFR+ and ER/HER2− is unclear. IBCMFs expressed higher p53 and proliferation markers in HR/HER2−; only 18.9% of IDCG3s had a basal‐like phenotype compared with 62.9% of IBCMFs. This is shown in Table 2 [83]. However, p53 expression was similar, with 78% of AMC and 69% of HGIDC. MC is a unique type of BC compared to AMC and HGIDC, and HER2‐neu amplification was seen in 46% of AMC and 56% of HGIDC [84]. Additionally, poorly differentiated and high proliferation tumors were substantially associated with overexpression of c‐myc protein but not LNMs [85], whereas MC was linked with cyclin E levels, and 433 patients with stroma‐enriched initial tumors found CCNE1 [86]. However, comparison with IDC and MBC exhibits greater accumulation of p53. Seventy‐one patients received treatment, and the tumor size was 25 mm, and the median age was 51 years. In this study, 33 out of 58 patients had p53 accumulation. This is shown in Table 3 [124]. Moreover, BC tumors with high histologic grade and weak differentiation are called MBC, and 20%–40% of IBT have p53 gene alterations, and it is present in MBC. About 20% of BCs have p53 mutations, and 30%–40% of tumors have p53 accumulation [125]. However, BLCs and HER2+ carcinomas are the most aggressive clinical behaviors of BC. Lower PTEN DNA copy numbers were found in BLCs, and a strong correlation between PTEN protein and PTEN DNA copy number [126]. Moreover, BCs resemble basal tissue and are linked to hereditary BRCA1 mutations and expression of EGFR and high cytokeratins, and basal‐like cancers are found to lack malfunctioning BRCA1 [127].

### 2.34. miRNA Signatures and Metastasis in Medullary Like Carcinoma

Most infectious agents implicated in the development of BC are HPV and EBV. miR‐143/145 downregulation is linked to HPV infection with promotion of metastasis status. This is shown in Table 4 [170]. Consequently, miR‐155 activity is suppressed by BRCA1, and the downregulation of BRCA1 by miR‐15/105/182 disrupts DNA repair and might alter BC treatment. TNBC and basal‐like sporadic BC cases arise from BRCA1 silencing miR‐146a and miR‐146‐5p. BRCA2 mutations may benefit from miR‐21‐focused therapy [171]. Besides, it was anticipated that miR‐199a‐3p would target the TSG gene BRCA1. TNBC cells have been treated with miR‐199a‐3p to target BRCA1, leading to its downregulation and decreased luciferase reporter activity mediated by BRCA1 3 ^′^‐UTR, and miR‐199a‐3p inhibited TNBC [172]. Therefore, miR‐622 is downregulated in BC, and this incident is linked to advanced grade and elevated Ki67 [173]. Additionally, three miRNAs, such as miR‐145‐5p, miR‐191‐5p, and miR‐21‐5p, were elevated in MBC [174].

### 2.35. Clinical and Preclinical Study of Different Molecularly Similar BC Subtype Treatments With Synthetic Drug Treatment Alone and/or in Combination

**Figure 3:**
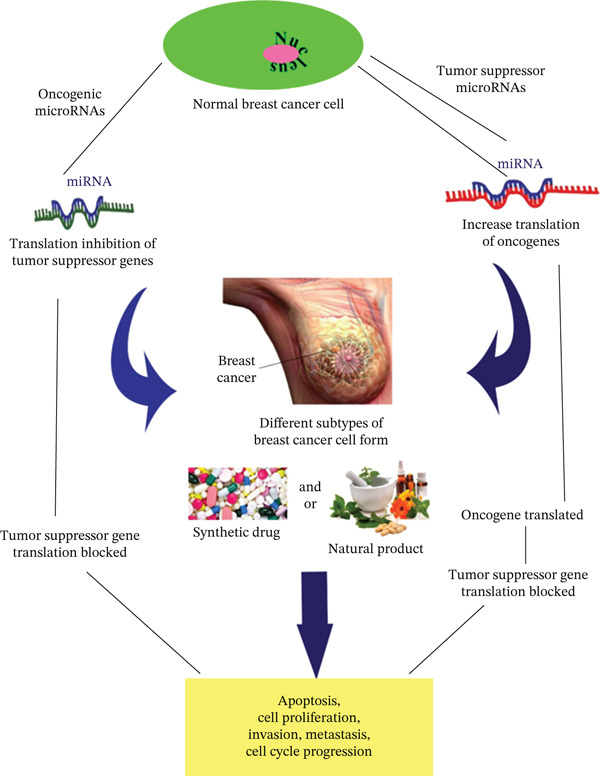
Targeted therapy of potential and natural drug activity on different breast cancer cell lines and overall activity of microRNAs, oncogenes, and tumor suppressor genes.

CDK4/6 and Er*α* signaling overexpression activated and made ER+ BC resistant to tamoxifen treatment, and LEM4 overexpression sped up the development of tumors. CDK4 and Rb are stabilized by interaction with LEM4, and Rb phosphorylation also encourages a G1/S phase transition. Tamoxifen resistance reverses with LEM4 reduction [209]. Moreover, evarolimus and paclitaxel had respective IC50 values of 32.50 and 7.80 *μ*g/mL, and mTOR expressions were lowered by evarolimus, and combination therapy is more effective. PI3K, p‐PI3K, and p‐AKT expression levels were reduced by paclitaxel, and their efficiency increased in combination therapy. This is shown in Table 5 and Figure 3 [210]. However, CEC was steady for 14 patients, about 74%, and CTC dropped in 22 patients, with 92% [211]. Therefore, tumor and/or ctDNA PIK3CA mutations were found in 40% of patients, and PFS was higher for tumor/ctDNA mutations than without, and PFS was longer with a normal metabolic state than in diabetes patients [212]. Consequently, miR‐26a expression was higher in HER2+ patients with 48 years of premenopausal therapy with docetaxel/trastuzumab [213]. Besides, miR‐26a and miR‐30b are induced by trastuzumab, and transfected BC cell proliferation was suppressed by 40% and 32%. Cell cycle study revealed that these miRNAs caused G1 arrest in HER2+ BC with trastuzumab treatment [214]. Additionally, patients with cardiotoxicity have decreased let‐7f, miR‐20a, miR‐126, miR‐210, and miR‐378, where cTnl expression decreased let‐7f, miR‐126, miR‐210, and miR‐378, whereas NT‐proBNP level is inversely connected with let‐7f and miR‐130a [215]. On the other hand, PI3KCA mutations in 36% of patients were linked to decreased miR‐549a, miR‐644a, and miR‐16‐5p, while drug treatment increased miR‐603, miR‐181a‐5p, miR‐199a, and miR‐199b‐3p greater than miR‐520d‐3p and miR‐548g‐3p [216], whereas BRCA1 wild‐type cells treated with gemcitabine alone and in conjunction with a PARP1 inhibitor showed increased expression of miR‐26a, ‐29b, ‐100, and ‐148a. This treatment helps to decrease miR‐206 expression, where there is an increase in BRCA1‐mutated cell treatment [217]. Furthermore, the tucatinib combination had higher clinical CNS‐PFS and ORR‐IC than the placebo combination and a 45.1% lower risk of acquiring new brain lesions than the tucatinib combination [218]. Although a strong basal‐like signature with high expression of keratins 5/17, cadherin 3, frizzled, and apolipoprotein D were characteristics of clustering of low expression of ESR1, while ESR1, GATA binding protein 3, and NAT were substantially expressed in LETS [219]. Besides the Ventana PDL1 assay, three to six patients who saw clinical benefit on atezolizumab/carboplatin had PDL1‐negative illness, whereas two to three patients with PDL1‐positive disease had progressing disease [220]. Therefore, letrozole treatment created a significant link between central scarring and clinical tumor volume decrease in 31 instances compared with two cases in chemotherapy [221]. However, lapatinib treatment was beneficial for patients with HER2‐negative/HER2‐enriched illness, while combined with endocrine treatment, lapatinib may be beneficial for HR+/HER2− illness that has a HER2‐enriched phenotype [222]. Although 35 days of three cycles of high‐dose cyclophosphamide, thiotepa, and docetaxel treatment gradually increased subsequent cohorts. Eleven patients finished three of 54 treatment cycles, and 44 BC patients experienced mucositis with diarrhea and interstitial pneumonitis, which were hazards that limited dosage [223]. Furthermore, trastuzumab‐treated patients having TP53 mutations and luminal A/B and TNBC patients have negative TP53 mutations, while patients receiving trastuzumab had highly favorable p53 immunopositivity [224]. Whereas 5813 patients received a BC diagnosis, and six received ACC treatment, while one patient had external beam radiation and HR therapy with tamoxifen [225]. However, the immune response is crucial for halting the growth of cancerous cells, where TILs are fundamental immune system elements. TLS and pCR were substantially correlated, and HR and TLS were independent predictors of pCR [226]. In addition, the effectiveness of maintenance immunotherapy in patients with metastatic HER2− IBC and TNBC, where a Phase II study of pembrolizumab monotherapy was conducted after induction chemotherapy, showed that pembrolizumab treatment with higher T‐cell clonality had higher PFS than lower baseline T‐cell clonality [227]. Furthermore, after advancing the CDK4/6 inhibitor, 111 patients were given evarolimus with ET, and patients without visceral metastases and those who have not had chemotherapy in the context of metastatic disease should get CDK4/6 inhibitor treatment for 18 months [228]. Hence, miR‐603, miR‐181a, miR‐199a, and miR‐199b‐3p expression was higher in 18‐month PFS patients than miR‐520d‐3p and miR‐548g‐3p. Low miR‐548g‐3p and miR‐520d‐3p may trigger Wnt/Hippo signaling, whereas miR‐603, miR‐181a‐5p, miR‐199a, and miR‐199b‐3p may suppress endocrine resistance and enhance treatment [229]. Therefore, PI3K‐AKT is an important biological function in BC, and miR‐200b, miR‐135b, and miR‐29a were shown to be trastuzumab‐resistant serums compared with trastuzumab‐sensitive individuals, whereas miR‐224 was found to be downregulated [230]. However, TQFL13 influences BC progression by modulating key cellular processes like growth, invasion, migration, and apoptosis. In vivo analysis TQFL13 has lower toxicity than TQ and is associated with downregulating PARP, BCL‐2, cyclin B1, cyclin D1, and p53 and upregulating BAX [231]. Adding to this, TQFL28 enhanced cytotoxicity and antiproliferative effects against TNBC, BT549, and 4T1, compared to TQ, and its IC50 was lower than TQ only, and in vitro analysis suppressed growth, migration, and invasiveness, and in vivo analysis inhibited tumor progression and metastasis [232]. In addition to this, pretreatment of TNBC and TQFL19 was characterized, and its ADMET properties were predicted. It has stronger cytotoxic effects in vitro analysis and induced cell cycle arrest, and in vivo analysis found suppressed cell growth, migration, and metastasis [233].

**Table 5:** Findings of microRNAs, oncogenes, and tumor suppressor gene activities treated with potential and application as targeted therapy.

Name of drug	Cell line/breast cancer subtype	Targeted marker	Dose and administrative way	Result	Reference
Tamoxifen	All breast cancer subtypes	Cyclin D, ER*α*, and RB		Increase ER*α* DNA binding and transactivity	[209]
Paclitaxel and everolimus	MDA‐MB‐231	PI3K, p‐PI3K, and p‐AKT	Everolimus and paclitaxel 32.50 and 7.80 *μ*g/mL	Highly decrease record in PI3K, p‐PI3K, and p‐AKT	[210]
Paclitaxel and gemcitabine	Metastatic breast cancer	HER2	Paclitaxel (P) 150 mg/m^2^ gemcitabine (2000 mg/m^2^ Days 1 and 15 with a cycle each 28 days)	Poor prognosis	[211]
Alpelisib + nab‐paclitaxel	HER2‐negative metastatic breast cancer	PI3K/PTEN	Alpelisib (250, 300, and 350 mg) daily plus nab‐paclitaxel 100 mg/m^2^ administered on Days 1, 8, and 15 every 28 days	Investigating more metabolic status	[212]
Docetaxel/trastuzumab	HER2‐positive metastatic breast cancer	miR‐26a	Docetaxel = 75 mg/m^2^ and trastuzumab = 6 mg/m^2^	Biomarker monitoring	[213]
Trastuzumab	HER2±, MCF‐7, and MDA‐MD‐231	miR‐26a, miR‐30b, and cyclin E	100 and 200 mg trastuzumab	Induce cancer cell growth	[214]
Epirubicin/cyclophosphamide		let‐7f, miR‐17‐5p, miR‐20a, miR‐126, miR‐210, and miR‐378		Biomarker and induce cardiotoxicity	[215]
Fulvestrant	HR+/HER2−	miR‐549a, miR‐644a, miR‐16‐5p, let‐7c‐5p, miR‐520d‐3p, miR‐548g‐3p, miR‐603, miR‐181a‐5p, and miR‐199a‐miR‐199b‐3p	Fulvestrant was administered 500 mg im q28 days	Induce cancer resistance	[216]
Gemcitabine	Basal‐like/triple‐negative breast cancer and MDA‐MD‐231/436	BRCA1, miRNA‐26a, ‐29b, ‐100, and ‐148a		PARP1 inhibitor‐reduced miR‐206	[217]
Tucatinib, pertuzumab, and trastuzumab emtansine, capecitabine, placebo, and capecitabine	HER2+ metastatic breast cancer	HER2	Tucatinib (300 mg orally twice daily), placebo + trastuzumab 6 mg/kg intravenously, and capecitabine 1000 mg/m^2^ orally	Induce cancer invasion and progression	[218]
Anastrozole	Integrative clusters	ERBB2, HSD17B1, STS, ESR1 CKND1, and microRNAs	Anastrozole 1 mg daily for 15 weeks	Induce ER‐positive breast cancer invasion and proliferation	[219]
Atezolizumab and carboplatin	Invasive lobular breast cancer	ER*α*	12 cycles of weekly carboplatin plus 1200 mg of atezolizumab every 3 weeks starting at three cycles of platinum‐based therapy	Improve the checkpoint blocked in invasive lobular BC	[220]
Letrozole	Special histological subtypes	ER*α*	Letrozole 47 mg daily for 30 weeks	Downsizing the breast tumor and enabling subservient surgery	[221]
Letrozole and lapatinib	Intrinsic subtypes	HER2	Letrozole 2.5 mg and/or lapatinib ditosylate 1500 mg for 6 months	Improving intrinsic subtypes of breast tumor treatment	[222]
Cyclophosphamide, thiotepa, and docetaxel	Metastatic breast cancer	Cyclin D	Cyclophosphamide (4 g/m^2^), thiotepa (300 mg/m^2^), and docetaxel (100 mg/m^2^ for 35 days)	Recommend for Phase II/III and reduce tumor size	[223]
Trastuzumab	Medullary like carcinoma	PIK3CA and TP53		Reduce TP53 gene mutation	[224]
Tamoxifen	Adenoid cystic carcinoma			Effective on breast cancer cell than radiation therapy	[225]
Trastuzumab	Tubular carcinoma	PTEN		Effective in breast cancer treatment	[226]
Pembrolizumab	Mucinous carcinoma	HER2	Pembrolizumab 200 mg was administered every 3 weeks, progression, intolerable toxicity, or 2 years of pembrolizumab exposure	Response best durable treatment	[227]
Exemestane, fulvestrant, and tamoxifen	Secretory carcinoma	CGK4/6 HER2		Novel clinical trials design	[228]
Fulvestrant	Triple‐negative breast cancer	miR‐549a, miR‐644a, miR‐16‐5p, let‐7c‐5p, miR‐520d‐3p, miR‐548g‐3p, miR‐603, miR‐181a, and miR‐199a‐miR‐199b‐3p	500 mg in 28 days	Targeted therapy with miRNA and long benefit for breast cancer resistance	[229]
Trastuzumab	Intrinsic subtype	miR‐200b, miR‐135b, miR‐29a, and miR‐224		Predictive biomarker of miRNAs and therapy	[230]
TQFL13	BT549 and MDA‐MB‐231	↓ PARP, BCL‐2, cyclin B1, cyclin D1, p53, and ↑ BAX		Strong inhibition of growth, invasion, migration, cell cycle progression, and apoptosis induction	[231]
TQFL28	BT549, MDA‐MB‐231, 4T1, and MCF10A	↓ PARP, BCL‐2, cyclin B1, cyclin D1, p53, and ↑ BAX	IC50: BT549 (38.78 ± 1.589 * μ*M) and MDA‐MB‐231 (39.63 ± 1.598 * μ*M)	Stronger cytotoxicity and antiproliferative activity than TQ	[232]
TQFL19	4T1, BT549, and MDA‐MB‐231	↑ BAX, ↓ cyclin B1, cyclin D1, ↓ PARP, and BCL‐2	IC50 not specified	Suppressed growth, migration, and metastasis in vitro and in vivo	[233]

### 2.36. Clinical and Preclinical Study of Different Molecularly Similar BC Subtype Treatments With Natural Drug Treatment Alone and/or in Combination

BC cell lines found that curcumin treatment with IC50 values of 25 *μ*M in 48 h, while this treatment decreased ERK and its phosphorylation, enhanced the generation of ROS in both cell lines [234]. Additionally, curcumin has shown a strong capacity to overcome resistance to IGF‐1‐induced apoptosis and inhibit IGF‐1‐stimulated MCF‐7 cell proliferation. Curcumin increased IGFBP‐3 while decreasing IGF1 production. This is shown in Table 6 and Figures 2 and 3 [235]. Moreover, baicalin treated 35 downregulated and 92 upregulated miRNAs, and Has‐miR‐3911, Has‐miR‐504‐5p, Has‐miR‐30a‐3p, Has‐miR‐193b‐3p, and Has‐181b‐5p decreased, and Has‐miR‐15a, Has‐miR‐100, Has‐miR‐16, and Hsa‐let‐7c increased, which that proved that baicalin controls p53 signaling, DNA template transcription, and DNA template proliferation [236]. Subsequently, baicalin anticancer efficacy against gynecological malignancies is based on underlying pathways including PI3K/AKT/mTOR, NF‐*κ*B, MAPK/ERK, and Wnt/*β*‐catenin, and causative processes including angiogenesis, autophagy, and apoptosis [237], whereas capsaicin decreased the expression of CDK8, and MDA‐MB‐231 transduced with LV CDK8 siRNA showed reduced levels of CDK8 mRNA and protein [238]. Besides, another research has been conducted that baicalin reduced ATP pools, AMPK activation, and autophagy induction, SFN induced energy stress, where miR‐23b, miR‐92b, and miR‐381 decreased in three BCs, as well as 60 miRNAs were overexpressed and 32 miRNAs downregulated [239]. Hence, apigenin IC50 50 *μ*M in BC can inhibit 17*β* estradiol‐induced ER activation. When apigenin was added to HER2+ BC SKBR3 cells, HER2/neu expression decreased, and PARP was cleaved [240]. Furthermore, in TNBC, MDA‐MB‐468 cells upregulated TNF*α*, and apigenin decreased TNF*α*‐elevated genes, including CXCL10, C3, PGLYRP4, IL22RA, KMO, IL7R, ROS1, CFB, IKBKs, SLITRK6, and MMP13 [241]. On the other hand, ginsenoside RK1 has anti‐inflammatory, anticancer, and anti‐insulin resistance properties. Cell growth, colony formation, LDH release, and G0/G1 phase arrest were suppressed by RK1. The ROS/PI3K/AKT pathway was charged with RK1‐induced apoptosis, which was provided by pretreatment with Z‐VAD‐FMK, PI3K/Akt activator, or ROS NAC [242]. In addition to this, the RK2 treatment expression of miR‐3614‐3p was suppressed by siRNA deregulation of CFAP20DC‐AS1. Rh2 and miR‐3614‐3p downregulated target genes, BBX and TNFAIP3, whereas lncRNA increased the inhibition of CFAP20DC‐AS1 [243]. In vivo HER2/neu BMDC in combination with QS‐21 and anti‐PD‐L1 mAb showed smaller tumors and splenocytes with increased cytotoxicity [244]. However, QS‐21 treatment differentially expressed 346 genes, and upregulated DEGs are indicative of cytokine release, TNF signaling, COX‐2, immunological activation, and steroid production. Losses in CDC, CCN, CDKs, CENP, KIFs, and PLKs are reflected in downregulated DEGs [245]. Moreover, UA naturally occurring in fruits and vegetables causes G1/G2 arrest, controlling signal transduction pathways and causing intrinsic and extrinsic death in BC [246]. Additionally, UA decreased ABCC1, which is the target of miR‐186‐5p, and UA inhibited ZEB1‐AS1, which upregulated ABCC1 [247].

**Table 6:** Findings of microRNAs, oncogenes, and tumor suppressor gene activities treated with natural drug application as targeted therapy.

Name of natural product and type	Biochemical sources	Chemical structure	Cell lines	Targeted marker	Results	Reference
Curcumin (phenols)			Luminal A/B breast cancer and MCF‐7	HER2	Potential therapeutic options in breast cancer	[234]
Curcumin (phenols)			Intrinsic subtypes and MCF‐7	IGF‐1 and IGF‐2	Apoptosis and downregulator of IGF‐1	[235]
Baicalin (flavon)			Luminal A and B basal‐like/triple‐negative breast cancer and MCF‐7	Has‐miR‐6799‐5p, Has‐miR‐6126, Has‐miR‐4792, Has‐miR‐6848‐5p, miR‐3197, miR‐6779‐5p, miR‐654‐5p, miR‐3911, miR‐504‐5p, miR‐30a‐3p, miR‐193b‐3p, and miR‐181b‐5p	Downregulator of different signaling pathways	[236]
Baicalin (flavon)			MCF‐7	miR‐217 and PTEN	Antibreast cancer therapy and downregulator of different signalings	[237]
Capsaicin (alkaloids)			Triple‐negative breast cancer and MDA MB 231	CDK8/PI3K/Akt/Wnt/*β*‐catenin	Cell cycle regulator	[238]
Capsaicin (alkaloids)			MCF‐7, MDA‐MB‐231, and SK‐BR‐3	miR‐23b, miR‐92b, miR‐381, and miR‐382	Cell cycle arrest and use anticancer therapy	[239]
Apigenin (flavone)			ER± and HER2±	HER2	Inhibit breast cancer growth	[240]
Apigenin (flavone)			Triple‐negative breast cancer and MDA MB 231/468	TNF*α*	Downregulation of TNF*α*	[241]
Ginsenoside Rk1 (saponins)			Triple‐negative breast cancer and MDA MB 231	ROS/PI3K/Akt, Bax, cytochrome C, cleaved caspase 3, 8/9, and BCL‐2	Innovative drug	[242]
Ginsenoside Rk1 (saponins)			MCF‐7	miR‐3614‐3p	Antiproliferation	[243]
QS‐21 (saponins)			HER2±	HER2	Antitumor activity	[244]
QS‐21 (saponins)			Triple‐negative breast cancer and MDA MB 231	CDK E/D	Proinflammatory and cytocytic	[245]
Ursolic acid			MCF‐7 and MDA MB 231		Anticancer drug	[246]
Ursolic acid			Triple‐negative breast cancer and MDA MB 436/468	miR‐186‐5p	Treatment of DOX‐resistant breast cancer	[247]

### 2.37. Possible Explanation of Why BC Cell Line Application for Natural and Synthetic Drug Available Instead of Molecularly Similar BC Subtype Cell Treatment

BC subtypes that differ molecularly are crucial. It helps physicians to forecast the most successful treatment and the chance of recurrence. Although gene expression studies have demonstrated the molecular foundation of BC, it is still unclear if these molecular traits will have a definitive impact on the clinical therapy of BC. In addition to variability across BC subtypes, it may be revealed by the discovery of miRNAs and certain genes linked to disease, such as oncogenes and TSGs. This information may also be useful in the creation of more targeted medications for each subtype, leading to treatments and extending life expectancy. This type of research will most likely also aid in early detection of cancer, improving patient survival rates. Molecular categorization is helpful for focused therapy as well as prognosis. So, we need to be included in the standard histopathologic report [248]. Significant gaps remain in the study of these rare BC subtypes despite significant advancements. These include the need for more sophisticated diagnostic techniques and a better understanding of their distinct biology, clarification of their immune and TME characteristics, discovery of new therapeutic targets, and revelation of molecular mechanisms of progression. Moreover, it is difficult to accurately diagnose these entities histologically due to their rarity, which emphasizes the need for effort to improve diagnostic criteria and maximize the use of auxiliary tools, as is done with ILC. However, by training AI models using a genetic ground truth rather than a histologic one, it may be possible to exploit the genotypic–phenotypic correlations that define a fraction of these rare BC subtypes to build AI‐based algorithms for their detection, and foundation models might be used to enhance the diagnosis of these uncommon entities by enabling the creation of strong AI techniques with a smaller sample. Therefore, new postmortem research initiatives that enable the gathering of large numbers of samples may offer important new information on the progression, dormancy, tumor heterogeneity, treatment resistance, and interactions with TME of these rare forms of BC. Finally, there is potential for improving precision medicine in these rare and distinct entities through subtype‐specific BC clinical trials, such as examining synthetic lethal approaches in ILC or those concentrating on young women′s BC with consideration for parity status for PPBC identification, whereas there are a lot of difficulties because there are not many situations for these rare entities [249]. The accumulation of these examples requires multi‐institutional initiatives. Research of these rare BC subtypes would be facilitated by the sharing of biospecimens and/or the creation of centralized repositories for clinicopathologic and genetic data. For this reason, it is essential that data collection and biomaterial handling practices be standardized across institutions. In addition to this making it easier to identify molecular drivers that may be used as therapeutic targets and possibly benefited from in consortium trials, multi‐institutional collaboration would enable the creation and application of AI tools for diagnosis and investigation of these entities. Attempts to improve diagnosis methods and treatment strategies for these unique subtypes of BC, as well as diligent attempts to comprehend their biology and molecular foundations, have the potential to improve patients′ outcomes. Next, there is a growing need to investigate individuals with BC subtypes because of their limited use. Scientists worldwide must have studied in vitro models of BC subtypes. Oppositely, molecularly well‐characterized cancer cell lines are great models for studying miRNAs, TSGs, oncogenes, key genes, altered cellular pathways in cancer, and evaluating anticancer drugs. We still do not fully understand this model′s methylation of DNA despite having an understanding of its genome. Before using any cancer cell line in a research program, molecular profiling is a must, and modern technologies are highly helpful for this. Given that epigenetic changes can take place early in oncogenesis and the DNA methylation pattern is a promising target of chemotherapy, it is imperative to explore the DNA methylation in cancer utilizing cell models. Subsequently, profiling molecular cancer cell lines is also crucial for the creation of novel anticancer medications as well as for comprehending patterns and modes of action of cell resistance to chemotherapeutics that are already utilized to treat cancer [250], unless profiling cancer cell lines can be an effective method for identifying potential therapeutic targets as well as changes in genes or pathways linked to genes or pathways linked to cancer.

## 3. Discussion With Future Perspective

BC continues to pose a significant clinical challenge due to its molecular heterogeneity and frequent development of therapy resistance. Detailed characterization of BC molecular subtypes and their subtype‐specific therapeutic options enables clinicians to personalize treatment strategies, reduce unnecessary toxicity, mitigate resistance, and improve overall patient outcomes. Beyond BRCA1/2, several tumor suppressor genes contribute to BC subtype diversity and progression. Alterations in PTEN, CHK2, ATM, and Rb disrupt PI3K/Akt signaling, DNA damage response, and cell cycle control, influencing tumor behavior and prognosis, while the proposed BRCA3 locus highlights the likelihood of additional, yet‐unidentified genetic drivers relevant to BC subtyping. c‐MYC oncogene, located at chromosome 8q24, is amplified or overexpressed in 12%–25% of BC and is often associated with aggressive tumor features and poor prognosis. Its dysregulation contributes to subtyping to subtype‐specific behavior, including hormone responsiveness and chemotherapy resistance, making MYC a potential therapeutic target despite ongoing debate about its role as a sole driver of breast carcinogenesis. PRR14 is markedly overexpressed in BC and, beyond activating PI3K/AKT/mTOR signaling, appears to modulate the ATM‐CHEK2 pathway in a BC‐specific manner. By suppressing CHEK2 and altering G2/M cell cycle control in a p53‐dependent context, PRR14 promotes tumor progression and is associated with poor chemotherapy response, suggesting its relevance as a subtype‐associated oncogenic driver and potential predictive biomarker. miRNAs represent promising therapeutic targets in BC through inhibition of oncogenic miRNAs or restoration of tumor‐suppressive miRNAs. Notably, miR‐21, miR‐10b, and miR‐34a show strong subtype‐specific relevance, with miR‐34a demonstrating antimetastatic and antiproliferative effects particularly in TNBC, underscoring the importance of evaluating miRNA‐based strategies within clearly defined BC subtypes. Although miR‐143 has emerged as a tumor‐suppressive miRNA across multiple BC subtypes, including TNBC and mucinous carcinoma, its downstream targets and context‐dependent regulatory mechanisms remain insufficiently defined. Moreover, translating miR‐143 into a therapeutic strategy faces challenges related to efficient delivery, stability, and sustained activity in vivo, highlighting the need for further mechanistic and translational studies before clinical application [251]. The study of oncogenes, tumor suppression genes, and miRNAs is critical for BC subtyping because these molecular regulators define the biological behavior, therapeutic response, and prognosis of each subtype. Oncogenes, when overexpressed or mutated, drive uncontrolled proliferation and contribute to aggressive phenotypes, helping distinguish high‐risk subtypes such as HER2‐enriched or MYC‐amplified tumors. Tumor suppressor genes, including BRCA1/2, PTEN, and CHEK2, regulate DNA repair, apoptosis, and cell cycle checkpoints; their loss or mutation not only informs subtype classification but also predicts susceptibility to targeted therapies like PARP inhibitors. miRNAs provide an additional layer of regulation, modulating oncogene and tumor suppressor networks in a subtype‐specific manner, influencing metastasis, hormone responsiveness, and chemotherapy resistance. Together, integrating data from these molecular players allows for more precise and biologically relevant subtyping, enabling personalized treatment strategies, improving prognostic accuracy, and identifying novel therapeutic targets. This approach is particularly important in heterogeneous subtypes like TNBC or HER2− low tumors, where conventional markers alone may fail to capture underlying molecular complexity. However, advances in BC molecular biology have enabled multigene classifiers that complement traditional classification, improving subtyping, prognosis, recurrence risk estimation, and therapeutic selection. Integrating genomics, proteomics, and metabolomics into clinically driven, multidisciplinary trials is essential to translate molecular discoveries into personalized therapies and improve outcomes, underscoring the need for continued research on metastasis, recurrence, and targeted treatment strategies.

## 4. Conclusion

BC remains a heterogeneous disease shaped by complex interactions among miRNAs, oncogenes, and TSGs. Our review highlights that while only a fraction of these regulators have been fully characterized, miRNAs are emerging as pivotal biomarkers for prognosis, diagnosis, and therapy prediction. Importantly, their coregulation with enhancers underscores a layered influence on both oncogenic and tumor‐suppressive pathways. Therapeutically, synthetic agents such as tamoxifen continue to demonstrate efficacy, while natural compounds, including baicalin and ursolic acid, show promise in modulating oncogenic signaling and expanding treatment options. Yet, the greatest obstacle lies in the validation of diagnostic and predictive miRNAs across independent cohorts and rigorous preclinical/clinical studies. The path forward demands targeted research into understanding BC subtypes with emphasis on unraveling their distinct physiological and pathological mechanisms. By integrating molecular insights with both synthetic and natural therapeutic avenues, the field can move closer to precise, personalized strategies that overcome relapse, metastasis, and resistance and the most pressing challenges in BC management today.

**Nomenclature**
LCIS: lobular carcinoma in situ
DCIS: ductal carcinoma in situ
IBC: inflammatory breast cancer
BC: breast cancer
ILC: invasive lobular carcinoma
TSGs: tumor suppressor genes
MBC: metastasis breast cancer
TNBC: triple‐negative breast cancer
ER: estrogen receptor
PR: progesterone receptor
PCR: polymer chain reaction
ISBC: intrinsic subtype breast cancer
IHC: immunohistochemistry
OS: overall survival
CTC: circulating tumor cell
ctDNA: circulating tumor DNA
CIN: chromosomal instability
CCND1: cyclin D1
CK18: cytokeratin 18
BLT: basal to luminal transition
PDA: poorly differentiated adenocarcinoma
TGF‐*β*
: transforming growth factor beta
TIL: tumor‐infiltrating lymphocytes
IntClus: integrative clustering
DWI: diffusion weighted imaging
MRI: magnetic resonance imaging
T2WI: T2‐weighted imaging
IDC: invasive ductal carcinoma
MED: medullary carcinomas
CGH: comparative genomic hybridization
FISH: fluorescence in situ hybridization
TC: tubular carcinoma
RB: retinoblastoma
NST: nonspecial type carcinomas
CVN: copy number variations
MC: mucinous carcinoma
ACC: adenoid cystic carcinoma
ROS: reactive oxygen species
SSBs: single‐stranded DNA breaks
DSBs: double‐stranded DNA breaks
RNS: reactive nitrogen species
GM‐CSF: granulocyte‐macrophage colony‐stimulating factor
IL: interleukin
NAC: N‐acetyl cysteine
DNA: deoxyribonucleic acid
RNA: ribonucleic acid
AHH‐1: human lymphocyte
LDH: lactate dehydrogenase
GSH: nonenzymatic antioxidant
SOD: enzymatic antioxidants
ATM: ataxia telangiectasia mutated
ATR: ataxia telangiectasia and Rad3‐related
DNA‐PK: DNA‐activated protein kinase
Chk: checkpoint kinase
CDK1: cyclin‐dependent kinase
CDC: cell division control protein
NBS1: Nijmegen breakage syndrome 1
NHEJ: nonhomologous end‐joining repair
HR: homologous recombination
DDR: DNA damage response
APE1: apurinic endonuclease 1
EGFR: epidermal growth factor receptor
RTKs: receptor tyrosine kinases
AKT: protein kinase B
PTEN: phosphatase and tensin homolog
mTOR: mammalian target of rapamycin
PI3K: phosphoinositide 3‐kinases
Apaf‐1: apoptotic protease activating factor 1
AIF: apoptosis‐inducing factor
BCL‐2: B‐cell lymphoma 2
Bax: BCL‐2 associated X
Bad: BCL‐2‐associated agonist of cell death
CDK4: cyclin‐dependent kinase 4
CDC2: cell division cycle 2
COX‐2: cyclooxygenase‐2
CYP19/1A1/1A2: cytochrome P45019/1A1/1A2
DNMT1/3a: DNA (cytosine‐5)‐methyltransferase 1/3a
DMBA: 9,10‐dimethyl‐1,2‐benzanthracene
ER*α*
: estrogen receptor‐ *α*
EGF: epidermal growth factor
EGFR: epidermal growth factor receptor
FoxM1: forkhead box M1
GSTA1: glutathione S‐transferase A1
HER2/3: human epidermal growth factor receptor 2/3
hTERT: human telomerase reverse transcriptase
JNK: jun N‐terminal kinase
LC3: microtubule‐associated protein light chain 3
MMP‐2: matrix metalloproteinase‐2
UTR: 3‐untranslated region
MDM2: mouse double minute 2 homolog
CCR6: C‐C chemoattractant cytokine receptor 6
ATF2: activating transcription factor‐2
RSU1: Ras suppressor‐1
PINCH1: protein containing five LIM domains
PIP2/3: phosphatidylinositol 4,5‐bisphosphate 2/3
VGFA: vascular endothelial growth factor A
HIF1: hypoxia inducible factor‐1
LNM: lymph node metastasis
CDH1: cadherin protein‐1
PIK3CA: phosphatidylinositol‐4‐5, bisphosphate 3‐kinase catalytic subunit alpha
PINK‐1: PTEN‐induced kinase‐1
MFN2: mitofusin‐2
CXCR4: C‐X‐C chemokine receptor type 4
SOD2: superoxide dismutase 2, mitochondrial
HUVEC: human umbilical vein endothelial cells
STX3: Shiga toxin 3
KLF16: Kruppel‐like factor‐16
PARP: poly ADP ribose polymerase
4EBP1: eukaryotic translation initiation factor 4E‐binding protein 1 phosphorylated
MAPK: mitogen‐activated protein kinases
NQO1: NAD (P) H quinone dehydrogenase 1
STAT3: signal transducer and activator of transcription 3
TrxR1: thioredoxin reductase 1
VEGFR‐2: vascular endothelial growth factor‐2
Mcl‐1: myeloid cell leukemia
FLIP: c‐FLICE inhibitory protein
Forkhead box O3: transcription factor FOXO3a
GSK3: glycogen synthase kinase 3
CSCs: cancer stem cells
EMT: epithelial–mesenchymal transition
Glut1: glucose transporter 1
TEAD: transcriptional enhanced associate domain
ceRNA: endogenous RNA
MBC: medullary carcinoma
ERK: extracellular single‐regulated protein kinase
HDACi: DNA methylation inhibitor
HPV: human papillomavirus
EBV: Epstein–Barr virus

## Author Contributions

Md. Sadikuj Jaman contributed to this manuscript in writing the original draft, supervision, conceptualization, methodology, data curation, figure design, and writing—review and editing. Md. Rokibul Hasan Bhuiyan and Md. Maniruzzaman contributed to methodology, writing the original draft, table arrangements, data collection and analysis, figure design and material preparation, and formal analysis. Md Shajedul Haque contributed to formal analysis, writing—review and editing, investigation, and conceptualization. Md. Sadikuj Jaman and Md. Maniruzzaman contributed equally to this work.

## Funding

No funding was received for this manuscript.

## Ethics Statement

The authors have nothing to report.

## Consent

The authors have nothing to report.

## Conflicts of Interest

The authors declare no conflicts of interest.

## Data Availability

Data sharing is not applicable to this article as no datasets were generated or analyzed during the current study.
